# Therapeutic effects of *Lactobacillus rhamnosus*, thymol and their combination against neurotoxicity in propionic acid (PA)-induced autistic rats: insights into the role of the Nrf2/HO-1, Wnt3/β-catenin/GSK3β BDNF/p-TrkB/CREB, pI3K/Akt/mTOR, AMPK/SIRT-1, and PERK/CHOP/Bcl-2 pathways

**DOI:** 10.3389/fphar.2025.1728908

**Published:** 2026-01-28

**Authors:** Hoda A. Salem, Nermin I. Rizk, Moustafa H. AbdelSalam, Rehab Ahmed, Hebatallah Husseini Atteia, Ahmed M. E. Hamdan, Areej A. Alghamdi, Manar A. Alghusn, Renad A. Alatawi, Rawan A. Atallah, Maryam M. Alfuhaymani, Hatun A. Alqahtani, Karema Abu-Elfotuh

**Affiliations:** 1 Department of Pharmacy Practice, Faculty of Pharmacy, University of Tabuk, Tabuk, Saudi Arabia; 2 Department of Physiology, Faculty of Medicine, University of Tabuk, Tabuk, Saudi Arabia; 3 Natural Products and Alternative Medicine Department, Division of Microbiology, Immunology and Biotechnology, Faculty of Pharmacy, University of Tabuk, Tabuk, Saudi Arabia; 4 Department of Pharmaceutical Chemistry, Faculty of Pharmacy, University of Tabuk, Tabuk, Saudi Arabia; 5 Prince Fahad bin Sultan Chair for Biomedical Research (PFSCBR), Tabuk, Saudi Arabia; 6 Faculty of Pharmacy, University of Tabuk, Tabuk, Saudi Arabia; 7 Department of Clinical Pharmacy, Faculty of Pharmacy, Al-Azhar University, Cairo, Egypt; 8 College of Pharmacy, Al-Ayen Iraqi University, An Nasiriyah, Iraq

**Keywords:** autism, autophagy, endoplasmic reticulum stress, oxidative stress, probiotics, thymol

## Abstract

**Background:**

Autism spectrum disorder (ASD) is a neurodevelopmental disease characterized by repetitive behaviors and a lack of social communication. The role of probiotics, phytochemicals and their combination phytochemicals as treatment options for ASD is still under study.

**Objective:**

This study aimed to evaluate the associated molecular pathways and explore the impact of *Lactobacillus rhamnosus* (*L. rhamnosus*), thymol (Thy) and their combination on propionic acid (PA)-induced ASD rats.

**Methods:**

Fifty 3-week-old male albino rat pups were randomly distributed into five groups. The groups included a control group, a PA-induced ASD group, in which PA (250 mg/kg, p.o.) was administered for 3 days, and three other groups that received PA (250 mg/kg, p.o.) for 3 days along with either *L. rhamnosus* (1 × 10^6 CFU/day, p.o), Thy (30 mg/kg/day, p.o), or both. Brain tissues were collected for biochemical, histological, and immunohistochemical studies following behavioral evaluations.

**Results:**

Compared with the group administered only PA, treatment with *L. rhamnosus*, Thy and their combination significantly improved the neurobehavioral deficits in the autistic group. Improvements were observed in tests assessing memory consolidation, learning capacity, attention, spatial memory, locomotor activity, and contextual information processing. In addition to histopathological improvements, *L. rhamnosus*, Thy and their combination demonstrated notable ameliorative effects on PA-induced abnormalities in brain neurotransmitters, oxidative stress, inflammation, apoptosis, and endoplasmic reticulum (ER) stress and autophagy biomarkers. Furthermore, treatment with *L. rhamnosus*, Thy and their combination improved abnormalities in the tested biomarkers and modulated associated pathways, including significant upregulation of BDNF, TrkB, CREB, Nrf2, and HO-1 content and downregulation of TLR4/NF-κB-mediated neuroinflammation, leading to substantial improvements in ASD symptoms.

**Conclusion:**

Our results suggest that *L. rhamnosus*, Thy and their combination have promising therapeutic potentials in alleviating biochemical and behavioral deficits in PA-induced autism. These effects may be mediated by halting apoptosis, inflammation, and endoplasmic reticulum stress, inducing autophagy, and improving different biomarkers and modulation pathways, such as Wnt3/β-catenin/GSK3β, pI3K/p-Akt/mTOR, and BDNF/p-TrkB/CREB.

## Graphical Abstract

**Figure:**
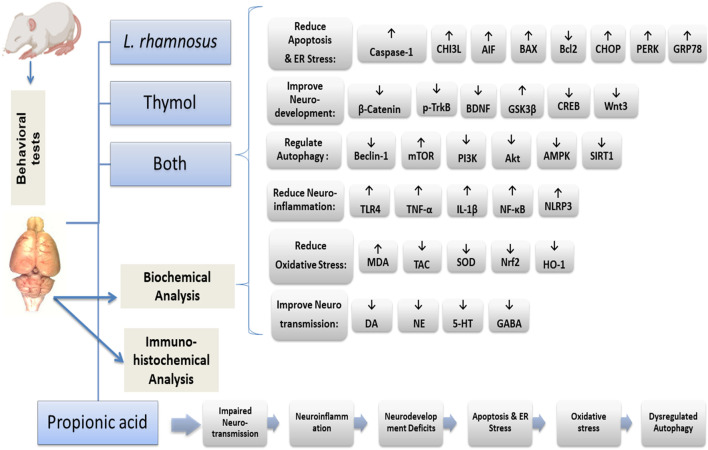
*L. rhamnosus is Lactobacillus rhamnosus*.

## 1 Introduction

The neurological disorder known as autism spectrum disorder (ASD) affects behavior, communication, and social relationships (Altomi et al., 2025). It has been proven that people with ASD have abnormal neurodevelopmental pathways that diverge from the normal patterns of brain development. The complex interaction of genetic factors, environmental effects, epigenetic mechanisms, cognitive processes, and behavioral components in ASD causes a wide range of symptoms and comorbid problems (Liloia et al., 2024).

Both genetic and non-genetic variables are probably involved in the complex etiology of ASD (Taylor et al., 2020; Sztainberg and Zoghbi, 2016; Sauer et al., 2021). Approximately 1% of people worldwide have ASD, with a higher male-to-female ratio (4:1) (Altomi et al., 2025). In addition to having intellectual disabilities, approximately 50% of people with ASD also frequently suffer from co-occurring neurodevelopmental and psychiatric disorders (Khogeer et al., 2022).

Oxidative stress and neuroinflammation are acknowledged as key factors influencing the development of ASD (Usui et al., 2023; Za et al., 2021). These processes frequently result in behavioral deficiencies, decreased neuroplasticity, and brain damage—all of which are characteristics of the pathophysiology of ASD. People with ASD frequently experience oxidative stress, which is caused by an excess of reactive oxygen species (ROS). Lipid and mitochondrial malfunction and an increase in malondialdehyde (MDA), a crucial indicator of oxidative damage, have all been linked to this imbalance in oxidative homeostasis. As demonstrated by increased pro-inflammatory cytokines such as prostaglandin E2 (PGE2), interleukin (IL)-6, IL-17, and tumor necrosis factor (TNF)-α, the ensuing oxidative stress may be a contributing factor to chronic neuroinflammation (Kılıç et al., 2025). Neurotrophic factors, such as brain-derived neurotrophic factor (BDNF), which are essential for synaptic plasticity and neuronal survival, are also downregulated by these inflammatory mediators (Kerschensteiner et al., 1999; Lai et al., 2018).

Numerous related proteins, including neurotransmitter transporters and receptors, are typically implicated in the clinical manifestation of various neurological conditions, including ASD. Deficits in ASD are linked to glutamate (Glu), dopamine (DA), serotonin (5-HT), and glutamate/GABA imbalance in the brain tissues (Bin-Khattaf et al., 2022; Quaak et al., 2013). Autophagy-related genes have exonic copy number variation mutations linked to ASD (Poultney et al., 2013). So, it is suggested that autophagy failure is a contributing factor to ASD. Through the IGF-1/PI3K/AKT/mTOR pathway, mammalian target of rapamycin (mTOR), a master regulator of cell proliferation, cellular metabolism, and autophagy, has been implicated in the development of ASD (Bozdagi et al., 2013; Zhang et al., 2020). In this scenario, it was suggested that autophagy is widely regulated through the PI3K/AKT/mTOR pathway, which will impact the development of ASD (Zhang et al., 2016). Moreover, it is reported that ASD progeny exhibited aberrant behavior that might have been caused by prolonged ER stress (Kawada and Mimori, 2018). Apoptosis, or programmed cell death, is a crucial process that controls the appropriate wiring of developing neural networks and shapes the brain’s size and structure. Neuroanatomic defects and developmental impairments can result from aberrant stimulation of apoptotic death mechanisms. Neural cell death has been shown to have a potential correlation with autism (Kılıç et al., 2025).

In order to simulate and investigate the pathogenic mechanisms underlying behavioral impairments and facilitate the search for a cure, animal models were developed. Chemical and genetic models are the two primary categories of animal models (Ali et al., 2022). The effectiveness of the chemical model depends on whether the substances utilized have the same effects on people. Propionic acid (PA) is a short-chain fatty acid that is formed regularly as a by-product of the metabolism of carbohydrates and certain sugars. It is detected in high concentrations in the blood, urine, and facial samples of autistic people (Choi et al., 2018; Cotrina et al., 2020) and is implicated in neurochemical autism. PA has been demonstrated to have a wide range of functions in host cellular physiology in both health and disease. Moreover, PA plays a vital role in immunity, metabolism, and development under healthy conditions, but it also impacts behavior and brain function in certain hereditary and acquired illnesses (Ma et al., 2011; Nankova et al., 2014; Abdelli et al., 2019). PA is a weak acid that is systematically absorbed by the gut, liver, and brain through passive and active monocarboxylate transporters. It also activates G-protein-coupled receptors (Ma et al., 2011). Developmental delay, oxidative stress, and metabolic or immunological abnormalities are associated with elevated PA levels; these symptoms share some parallels with those of propionic acidemia and autism (Cotrina et al., 2020; Ma et al., 2011; Nankova et al., 2014). Bacteria that produce PA, such as *Bacteroidetes*, *Clostridium*, and *Desulfovibrio*, are commonly found in high concentrations in the stomachs of people with ASD. In addition, PA is also frequently added to processed meals that contain carbohydrates, which many children with ASD consume (Thomas et al., 2012). This, in addition to gastric absorption, causes the gut microbiota to produce more of this acid (Ma et al., 2011).

The administration of PA has been found to prompt disruption, neuroinflammation, and oxidative stress of synaptic plasticity pathways, which is presented by a significant elevation of NF-kB, TNF-α, and MDA, a significant reduction in NRF2, CAT, SOD, and BDNF levels, and disruption of the Wnt/β-catenin pathway, making it an appropriate model to study autism and therapies that affect these mechanisms (He et al., 2018; Sahin et al., 2022).

In addition to its antibacterial, antioxidant, anticancer, anti-inflammatory, and antitussive properties, Thy is a naturally occurring phenolic monoterpene derivative of the umbelliferous hydrocarbon and carvacrol tautomer (Xiong et al., 2023). Additionally, Thy is a volatile oil with several benefits: low molecular weight, easy blood–brain barrier crossing, high volatility, and suitability for treating brain problems (Zhang et al., 2021). Thy has demonstrated effectiveness in blocking a variety of inflammatory indicators, including cyclooxygenase-2; interleukins IL-1β, IL-6, and IL-8; and tumor necrosis factor-alpha (TNF-α). Its ability to disrupt the NF-κB and MAPK pathways, which are key modulators of inflammatory gene expression, is primarily responsible for these effects. These molecular processes highlight the capacity of Thy to function in several clinical situations where either acute or chronic inflammation is important (Gago et al., 2025). Furthermore, Thy protects rats against monosodium glutamate-induced attention-deficit/hyperactivity disorder-like behavior (Abu-Elfotuh et al., 2022a). In the depression model of chronic unexpected mild stress, Thy may control the expression of TNF-a and IL-1β (Deng et al., 2015). The positive benefits observed after Thy therapy are largely due to the p38 MAPK pathway (Shu et al., 2016). These results show that Thy might control inflammation through the Pin1/p38 MAPK pathway in rats with ASD (Xiong et al., 2023).

*L. rhamnosus* is found in the native microbiota and is beneficial for health because it can reduce oxidative stress and possesses anti-inflammatory properties. It can also restore the gastrointestinal barrier function, alter the levels of several neurotransmitters and cytokines, and modulate different signaling pathways (Alsubaiei et al., 2022). Additionally, the primary metabolites of the microbiome, short-chain fatty acids (SCFAs), which are generated by bacterial fermentation, might influence the microbiome–gut–brain axis either directly or indirectly. The dosage of these metabolites may also be crucial in determining how they alter the behavioral and psychophysiological processes (Martin et al., 1984). Moreover, *L. rhamnosus*, which produces GABA, may be a suitable candidate to alter the glutamate/GABA ratio, making it a potentially effective treatment for social behavioral symptoms linked to ASD (Patel et al., 2020; Saber et al., 2021).

However, the role of probiotics and/or phytochemicals through the gut–microbiota–brain axis as treatment options is still under study. In this study, we hypothesized that *L. rhamnosus*, Thy and their combination would ameliorate PA-induced neurobehavioral deficits by modulating oxidative stress, neuroinflammation, and apoptosis and restoring key neurodevelopmental and synaptic plasticity pathways, and we analyzed our theory by testing behavioral and histopathological changes and the levels of specific biological markers.

## 2 Materials and methods

### 2.1 Drugs and dosage

Thymol (Thy) and all other chemicals used (analytical grade) were purchased from Sigma-Aldrich Chemical Co. (CAS No.: 89-83–8, St. Louis, MO., United States).

### 2.2 Bacterial strain and culture conditions

A standard strain of *L*. *rhamnosus* (ATCC 7469) was used in this study. It was cultured in 100 mL of liquid de Man, Rogosa, and Sharpe (MRS; OXOID Ltd., United Kingdom) agar (Oxoid, England) and broth media in a shaker incubator set to 200 rpm at 37 °C for 24 h under aerobic conditions (Zhang et al., 2020). A suspension of *L. rhamnosus* (1 × 10^6^ CFU) was prepared in sterile saline for feeding the rats. Briefly, a saline solution (0.9% sodium chloride) was used to resuspend the sediments. We combined the *L. rhamnosus* suspensions and added the physiological saline solution until the final amount was 10 mL. This mixture was prepared daily and used immediately. We periodically examined the sediment for the quantity and type of microorganisms before mixing it. We employed the double dilution approach and MALDI-TOF in this verification procedure (Zhang et al., 2016).

### 2.3 Animals

Male 3-week-old albino rat pups (n = 50) that were newly born and weighed between 25 and 30 g were used in the study. The animals were housed in controlled environments with a constant temperature of 24 ±1 °C and a light/dark cycle of 12/12 h. Rats were kept in stainless steel cages in a hygienic animal room under a regular light–dark cycle at 26 °C ± 4 °C. All animal procedures were carried out as previously described (Abu-Elfotuh et al., 2023). Briefly, rats were provided with standardized AIN-93 Purified Rat Diets (El-Nasr Company, Abu Zaabal, Cairo, Egypt) and water *ad libitum*.

### 2.4 Ethical consideration and approval

The NIH Guideline for the Care and Use of Laboratory Animals (NIH Publications No. 85–23, updated 2011) was followed. Moreover, all animal handling procedures were overseen by the Animal Ethics Committee of the Faculty of Pharmacy (Girls), Al-Azhar University, Egypt (No.384/2023). Every effort was made to minimize the suffering experienced by the animals throughout the trial.

### 2.5 Induction of autism spectrum disorder in rats

The rats were given freshly prepared PA (250 mg/kg) in double-distilled water orally via an intragastric tube for 3 days and were then administered daily oral saline for the remaining 27 days (until the end of the study period) (Alsubaiei et al., 2022).

### 2.6 Design of experiments

Following weaning, rat pups were randomly assigned to one of five groups (n = 10/group) using a computer-generated random number sequence. Each animal was given a temporary cage number. After assigning each rat to an experimental group, the temporary numbers were replaced with permanent, distinct, sequential, identification numbers. All animals were weighed and checked for clinical abnormalities by expert technicians on the final day of the quarantine period in order to exclude those deemed unsuitable for research. The animal weights offer the day 0 data and are an essential component of the randomization procedure. A suitable data gathering method was used to insert body weights and temporary cage numbers straight into a file or from paper tape or computer cards (Martin et al., 1984).

Based on standard procedures in comparable preclinical behavioral and biochemical studies using this model, a sample size of n = 10 per group was chosen to ensure sufficient statistical power to detect significant effect sizes. To guarantee an impartial distribution of litter effects across all groups, rat pups were randomly assigned to one of the five experimental groups (n = 10 per group) after the weaning period using a computer-generated random number sequence.

Fifty 3-week-old male albino rat pups were randomly divided into five groups.

The following groupings were allocated from the animals, with 10 animals in each group.

Group 1: control group; rats were given oral normal saline every day for 4 weeks.
Group 2: the autistic group; rats were given oral PA (250 mg/kg) for 3 days, followed by daily oral saline for the remaining 27 days.
Group 3: the PA + *L. rhamnosus* group; rats were given oral PA (250 mg/kg) for 3 days and then *L. rhamnosus* suspension (1 × 10^6 CFU/day) (Patel et al., 2020) orally for the remaining 27 days (Alsubaiei et al., 2022).
Group 4: the PA + Thy group; rats were given oral PA (250 mg/kg) for 3 days and then oral Thy (30 mg/kg/day) (Saber et al., 2021) every day for the remaining 27 days.
Group 5: the PA + *L. rhamnosus* + Thy group; rats were given oral PA (250 mg/kg) for 3 days and then both *L. rhamnosus* suspension (1 × 10^6 CFU/day) and Thy (30 mg/kg/day) by oral gavage (Nagoor Meeran et al., 2017) every day for the remaining 27 days (Abu-Elfotuh et al., 2023; Saber et al., 2021).

#### 2.6.1 Experiment timeline

Fifty male rat pups (3 weeks old) were split into five groups at random (n = 10). During the PA-induction phase, PA (250 mg/kg) was administered orally for 3 days in a row. Following this, groups were given daily oral dosages of saline accompanied with either *L. rhamnosus* (1x10^6 CFU), Thy (30 mg/kg), or their combination for a duration of 27 days. Saline was administered to the control group for the full period of 30 days. Following behavioral testing at the conclusion of the treatment period, brain tissues were obtained for immunohistochemical, molecular, and biochemical investigation.

All behavioral testing, biochemical tests, and histological/histopathological evaluations were carried out by expert technicians who were blinded to the animal and sample treatment group assignments in order to reduce bias.

### 2.7 Behavioral studies

After the end of the 30-day period (from the start to the end of the end of the experiments), the animals were transferred to the behavioral laboratory for a 1 -hour acclimation period before the start of behavioral testing, without access to food or water. Experiments were carried out at a fixed time between 9 a.m. and 2 p.m.

#### 2.7.1 Open-field test

The device used in these experiments was a wooden box that was square-shaped and had white flooring and crimson walls. The floor was separated into two sections, each measuring 80 cm in length and 40 cm in height, and divided into 16 identical 20 cm × 20 cm square sections (Ali et al., 2017). Each rat was positioned in the middle of the device. By tracking the latency time(s), which is the amount of time between dropping an animal and its choice to move, and the ambulation frequency, which is the number of crossed squares over a 3-min period, the behavioral parameters for locomotor activity were assessed (Isaev et al., 2020).

#### 2.7.2 Y-maze test

According to Hughes et al.’s experiment (Aspide et al., 1998), the Y-maze used was a wooden, black maze consisting of three equal-sized arms, designated A, B, and C. Each arm measured 12 cm in width, 40 cm in length, and 35 cm in height and was positioned at 120° away from the other two arms (Hughes, 2004). For 8 minutes, rats were placed at the end of one arm and given complete freedom to move throughout the maze. Entry into all three arms on consecutive choices was defined as spontaneous alternation behavior (SAB). SAB is a reflection of both attention and spatial memory (Aspide et al., 1998). The following formula was used to determine the percentage of spontaneous alternation: (number of alternations/total arm entries) X 100 = spontaneous alternation.

#### 2.7.3 Forced swimming test

The forced swimming test (FST) assesses the effectiveness of potential antidepressant medications (Teixeira et al., 2013). FST was conducted in a plexiglass cylinder that measured 30 cm in diameter and 45 cm in height. To keep a rat’s paw from reaching the plexiglass floor, the water was just 25 cm deep. Training and testing were applied to the two phases of this experiment. During the training phase, each rat spent 15 minutes in a swimming pool. The last 5 minutes were used to record the rat’s activity.

The amount of time that each rat is movable is recorded during the behavioral analysis. The immobility time is then calculated by deducting the overall mobility time from the 240 s of the test time.

Each rat was submerged in the water four times, and the data gathered corresponded with the training phase. Increased immobility time is interpreted as a depressive-like behavior (Can et al., 2012; Carlezon et al., 2002).

#### 2.7.4 Conditioned avoidance response test

The conditioned avoidance response (CAR) test is used to evaluate memory consolidation and learning capacity under extreme stress (Castagné et al., 2009). For this test, a wooden box apparatus measuring 43.3 × 11.8 × 15.8 inches was utilized. The box has moveable glass panels that separate it into five connected rooms. A stimulator set to 50 V and 25 pulses per second electrified the four rooms’ flooring, which was composed of a grid of parallel metal rods. The sixth chamber’s floor was composed of glass.

A day before testing, the experimental animals were trained. Five seconds of auditory stimuli (a conditioned stimulus) were paired with 5 seconds of foot shock to conduct the training. For 2 days, the same animals underwent repeated testing. The number of trials [on the first and second day] after treatment that each rat needed to complete in order to reach the safe region (and avoid the electric shock) during the 5 seconds of the conditioned stimulus prior to the electric shock being administered was determined.

Rats were weighed at the end of the experiments; following that, pentobarbital (50 mg/kg) was used to euthanize the rats, and the animals were terminated by cervical dislocation. After that, the brains were dissected right away, cleaned with ice-cold saline (0.9 percent w/v), and cut into three parts. The first portion was immediately frozen on ice and kept at −80 °C until it was used for polymerase chain reaction (PCR) analysis to evaluate gene expression. The second part was kept at −20 °C until it was utilized for biochemical studies. The third component was stored in the proper buffer until it was used for histopathological analysis (Abu-Elfotuh et al., 2022a).

### 2.8 Biochemistry analyses

#### 2.8.1 Enzyme-linked immunosorbents assay

The following biomarkers were assessed in 10% brain supernatant using rat-specific enzyme-linked immunosorbent assay (ELISA) kits and following the manufacturer’s instructions: the proteins include apoptosis-inducing factor (AIF), TNF-α, interleukin (IL)-1β, gamma-aminobutyric acid (GABA), dopamine (DA), Wingless-type MMTV Integration Site Family (Wnt3), β-catenin, glycogen synthase kinase-3 beta (GSK3β) (MyBioSource, Inc., San Diego, CA, United States, Cat # MBS163437, MBS175904, MBS2023030 MBS269152, MBS701755, MBS2025504, MBS261324, and MBS766198, respectively), and noradrenaline (NA) (Cusabio Technology, China, Cat. No. CSB-E07870m).

#### 2.8.2 Colorimetric assays

Using commercial colorimetric assay kits supplied by Bio Diagnostics, Inc., Giza, Egypt, a 10% brain supernatant was colorimetrically examined for total antioxidant capacity (TOC) and malondialdehyde (MDA).

#### 2.8.3 Gene expression measurement by quantitative real-time PCR analysis

Using quantitative real-time PCR (qPCR) analysis to quantify gene expression using Applied Biosystems StepOnePlus technology and qPCR, the research biomarkers and the housekeeping gene *β-actin* mRNA levels in the brain tissue were evaluated. First, the total RNA was extracted using a QIAGEN Tissue Extraction Kit®. A SensiFAST cDNA Synthesis Kit® (Cat. # BIO- 65053) was then used to reverse-transcribe the isolated mRNA (Alsubaiei et al., 2022). Primers for DNA amplification, QIAGEN® DNA Master Mix, and real-time qPCR SYBR Green® were used. Software version 3.1 (StepOneTM, United States) from Applied Biosystems® was used to evaluate the data in relation to the housekeeping gene, *β-actin*. Table 1 displays the primer sequences. All experiments were carried out in triplicate. The primers were produced by Shanghai Jierui Biological Engineering Co., LTD., after being chosen from the PubMed database. The relative gene expression (^2−ΔΔCT^) method was used to assess the RT-PCR data (Abu-Elfotuh et al., 2022a).

**TABLE 1:** Primer sequences utilized in the qPCR analysis of rat brain tissues.

Gene	Forward primer	Reverse primer	GenBank accession number
*AIF* *AKT1* *AMPK* *Bax* *Beclin-1* *Bcl2* *caspase-1* *CREB1* *CHOP* *GRP78* *H O -1* *mTOR* *NF-κB* *NLRP3* *Nrf2* *PERK* *pI3K* *TrkB* *SIRT1* *TLR4* *β-actin*	5′-CCGGCTTCCAGGCAACTTGTTCC-3′ 5′-TCACCTCTGAGACCGACACC-3′ 5′− AAAGAACCCTAGCCTGAAGAGG-3′ 5′-GTTGCCCTCTTCTACTTTG-3′ 5′-AGCACGCCATGTATAGCAAAGA-3′ 5′-CGGGAGAACAGGGTATGA-3′ 5′-GAACAAAGAAGGTGGCGCAT-3′ 5′-TGGAGTTGTTATGGCGTCCTC-3′ 5′-TCTGCCTTTCGCCTTTGAG-3′ 5′-GACATCAAGTTCTTGCCGTT-3′ 5′-CACCAGCCACACAGCACTAC-3′ 5′-GGGCGTAGCGATAATGGAG-3′ 5′-TTCCTCAGCCATGGTACCTC-3′ 5′-TGCATGCCGTATCTGGTTGT-3′ 5′-CTCTCTGGAGACGGCCATGACT-3′ 5′-GCCGATGGGATAGTGATG-3′ 5′-GCCCAGGCTTACTACAGAC-3′ 5′- CCTCCACGGATGTTGCTGA-3′ 5′- GGCACCGATCCTCGAACAAT-3′ 5′-TCAGCTTTGGTCAGTTGGCT-3′ 5′-CCGTAAAGACCTCTATGCCA- 3′	5′-CCCGGATGGATCTAGCTGCTGCA-3′ 5′-ACTGGCTGAGTAGGAGAACTGG -3′ 5′-ACCTTCCGAGATGAATGCTTTT-3′ 5′-AGCCACCCTGGTCTTG-3′ 5′-GGAAGAGGGAAAGGACAGCAT-3′ 5′-CAGGCTGGAAGGAGAAGAT-3′ 5′-GAGGTCAACATCAGCTCCGA-3′ 5′-AACCTCTCTCTTTCGTGCTGC-3′ 5′-GCTTTGGGAGGTGCTTGTG-3′ 5′-CTCATAACATTTAGGCCAGC-3′ 5′-CACCCACCCCTCAAAAGACA-3′ 5′-TGCCGTCATCTGTCTTTCC-3′ 5′-ACCTCTTGCGAGGGTCTTTG-3′ 5′-ACCTCTTGCGAGGGTCTTTG-3′ 5′-CTGGGCTGGGGACAGTGGTAGT-3′ 5′-GCTTTGGGAGGTGCTTGTG-3′ 5′-AAGTAGGGAGGCATCTCG-3′ 5′-GGCTGTTGGTGATACCGAAGTA-3′ 5′-CGCTTTGGTGGTTCTGAAAGG-3′ 5′-GTCCTTGACCCACTGCAAGA-3′ 5′-AAGAAAGGGTGTAAAACGCA- 3′	AF375656 NM_033230.3 NM_023991.2 NM_031530 NM_001034117.1 NM_199267.2 NM_012762 NM_134443.2 NM_001109986.1 NM_013083.2 NM_012580 NM_019906.2 NM_001276711.2 NM_001191642 NM_031789 NM_001313915.2 NM_001371300.3 NM_001163168.3 NM_019178 NM_019178.2 NM_031144

### 2.9 Immunohistochemical examinations

Brain tissue specimens were preserved in buffered neutral formalin (10%) for histopathological analyses. After being dehydrated, hippocampal specimens were sectioned (4 µm–5 µm) and embedded in paraffin. Hematoxylin and eosin (H&E) were used to stain the sections, which were then analyzed using a Nikon Eclipse E200-LED microscope (Tokyo, Japan, ×200 and ×400 magnification). A semi-quantitative scoring system was used to characterize the extent of hippocampal damage (degree of nuclear pyknosis and degeneration and brain tissue lesions). Five grades were assigned based on the degree of injury: 1, minimal injury (<1%); 2, slight injury (1%–25%); 3, moderate injury (26%–50%); 4, moderate/severe injury (51%–75%); and 5, severe injury (76%–100%) (Nakaji et al., 2002).

Brain tissue sections were treated with an anti-glial fibrillary acidic protein (anti-GFAP) antibody (1:800 dilution; Servicebio, United States, Cat# GB12090) at 4 °C for a whole night in order to detect glial fibrillary acidic protein (GFAP). This was followed by a 1-h incubation with a biotinylated secondary antibody at room temperature. Brown precipitate was developed using the 3,3-diaminobenzidine peroxidase substrate kit. A Nikon Eclipse E200 LED (Tokyo, Japan) was used to view the photos at a magnification of ×400 (Albrakati et al., 2021).

### 2.10 Analysis of statistics

Data were expressed as the mean +S.E.M., and statistical analysis was carried out using one-way ANOVA followed by Tukey’s multiple comparisons test to determine the significance of differences between treatments. Values of *p* < 0.05 were considered significant. All statistical analyses were performed, and graphs were sketched using GraphPad Prism (ISI, United States) software (version 6).

## 3 Results

In this work, the actions of *L. rhamnosus* and/or thy on normal control groups were studied; however, these three groups showed no significant differences from the normal control group in all the measured biochemical parameters or in the histopathological findings. Consequently, the actions of *L. rhamnosus*, Thy and their combination on normal rats are not shown in order to avoid the complexity of data.

### 3.1 Therapeutic effects of *L. rhamnosus*, Thy and their combination on the behavioral changes detected in PA-induced neurotoxicity

**FIGURE 1:**
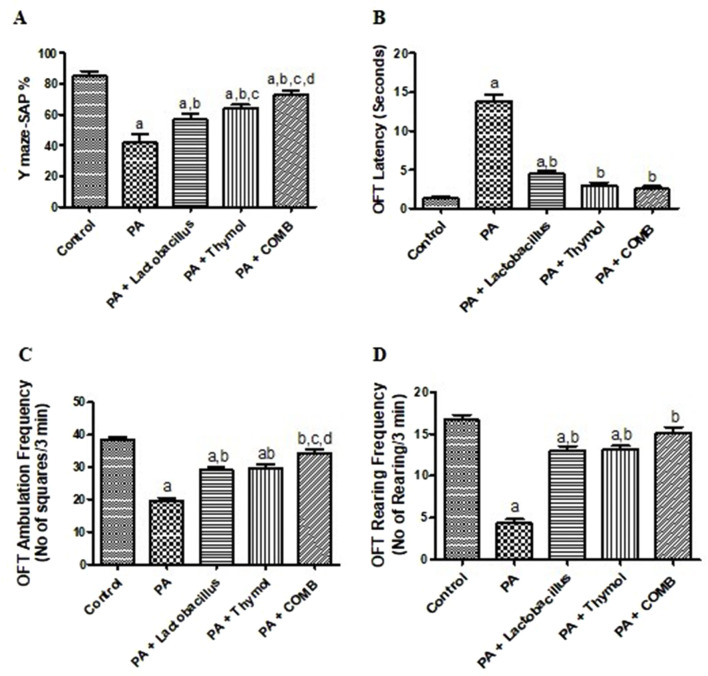
Therapeutic effects of *L. rhamnosus*, Thy and their combination on the behavioral changes detected in PA-induced neurotoxicity; Y-maze test: **(A)** SAP (%); OFT tests: **(B)** latency duration, **(C)** ambulation frequency, and **(D)** rearing frequency. **(a)** Significance relative to the control group. **(b)** Significance relative to the PA group. **(c)** Significance relative to PA + *Lactobacillus rhamnosus*. **(d)** Significance relative to PA + Thy. PA, propionic acid, OFT, open-field test, SAP, spontaneous alternation performance. The mean ± SEM was used to establish the results (n = 6). One way ANOVA test was used, followed by Tukey’s *post hoc* test, and the represented *p*-value is <0.05.

In the Y-maze test, an indication of spatial working memory and cognitive function, administration of PA significantly reduced SAP% by 49.2% compared to that in normal control rats (Figure 1A). Treatment with *L. rhamnosus*, Thy and their combination significantly increased SAP% by 26.8%, 35%, and 43%, respectively, compared to that in the PA group. In the same way, in the open-field test, an indication for the locomotor activity and anxiety-like behaviors showed that administration of PA caused disarray in decision-making by significantly prolonging the latency score to approximately 10.6 times, decreasing the attentive processes, ambulation, and rearing frequencies that underlie the gathering of contextual information in novel situations, as evidenced by a significant decrease in rearing frequency to 25.7% and decreasing locomotor activity to 50% compared to those in the control group.

These behavioral changes were considerably lessened upon treatment with *L. rhamnosus* (32.6% decrease and 3-fold and 1.5-fold increase for the latency score, rearing, and ambulation frequencies, respectively); with Thy (21.7% decrease and 3.1-fold and 1.5-fold increase for the latency score, rearing, and ambulation frequencies, respectively); and with both *L. rhamnosus* and Thy (18% decrease and 3.5-fold and 1.7-fold increase for the latency score, rearing, and ambulation frequencies, respectively) compared to that in the PA group. Interestingly, the measured behavioral parameters returned to their normal values through co-administration of both *L. rhamnosus* and Thy (Figures 1B–D).

More detailed values of the results are illustrated in Supplementary Tables 1–4.

**FIGURE 2:**
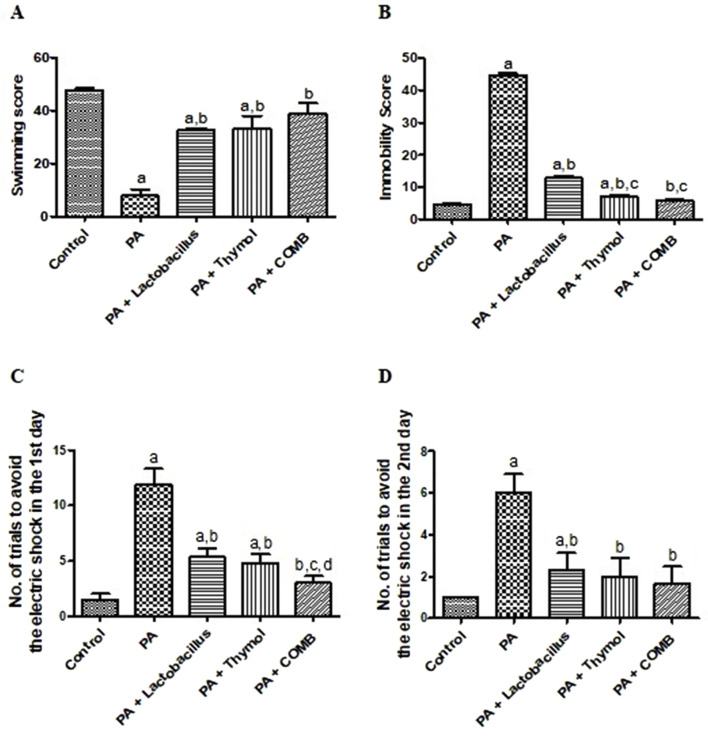
Therapeutic effects of *L. rhamnosus*, Thy and their combination on behavioral changes induced by PA-related neurotoxicity. **(A)** Swimming, **(B)** immobility in the FST, and performance in the CAR behavioral test. **(C)** No. of trails to avoid electric shock in the 1st day. **(D)** No. of trails to avoid electric shock in the 2nd day. **(a)** Significance relative to the control group. **(b)** Significance relative to the PA group. **(c)** Significance relative to the PA + *Lactobacillus rhamnosus* group. **(d)** Significance relative to the PA + Thy group. Significance: *p* < 0.05. PA, propionic acid. The mean ± SEM was used to establish the results (n = 6). One way ANOVA test was used, followed by Tukey’s *post hoc* test, and the represented *p*-value is <0.05.

In the FST behavioral tests, for the swimming score (Figure 2A), the PA group showed a significant reduction of 83.3% compared to the control group. Meanwhile, administration of *L. rhamnosus*, Thy, or their combination showed significantly increased swimming scores by 4.1, 4.2, and 4.9 times, respectively, compared to that in the PA group. Interestingly, the *L. rhamnosus* and Thy combination group showed no significant difference in the swimming score compared to that in the control group. In the same way, administration of PA considerably increased the immobility score by 9.5 times compared to that in the control group. Meanwhile, the immobility scores of rats improved significantly with the administration of *L. rhamnosus*, Thy, or their combination compared to those in the PA group (Figure 2B). Interestingly, the *L. rhamnosus* and Thy combination group had the highest efficacy, returning the scores to the normal level without a significant difference compared to those in the control group.

Concerning the conditioned avoidance response test (CAR), compared to the control group, the PA group showed an increase in the avoidance response number to the electric shock by 7.7 times on the first day (Figure 2C) and 6.5 times on the second day (Figure 2D). The difference between the two groups was statistically significant on both the first and second days. The number of trials for electric shock avoidance response decreased by approximately 58.3%, 60.9%, and 74%, respectively, on the first day and by approximately 61.5%, 69.2%, and 73.8%, respectively, on the second day after treatment with *L. rhamnosus*, Thy, or their combination compared to that in the PA group. Interestingly, in the same way, the *L. rhamnosus* and Thy combination group still had the highest efficacy for the electric shock avoidance response, returning the scores to the normal level without a significant difference compared to that in the control group.

### 3.2 Therapeutic effects of *L. rhamnosus*, Thy and their combination on the cerebral levels of neurotransmitters in PA-induced neurotoxicity

**FIGURE 3:**
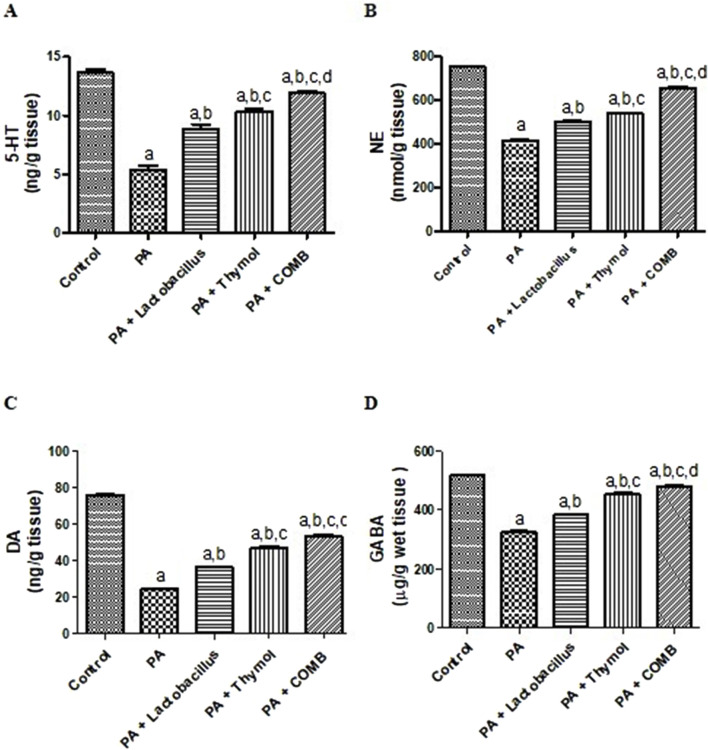
Therapeutic effects of *Lactobacillus rhamnosus*, Thy and their combination on the cerebral levels of neurotransmitters in PA-induced neurotoxicity. **(A)** 5-HT, **(B)** NE, **(C)** DA, and **(D)** GABA in autistic rats. **(a)** Significance relative to the control group. **(b)** Significance relative to the PA group. **(c)** Significance relative to the PA + *Lactobacillus rhamnosus* group. **(d)** Significance relative to the PA + Thy group. Significance: *p* < 0.05. PA, propionic acid. 5-HT, serotonin; NE, norepinephrine; DA, dopamine; GABA, gamma-aminobutyric acid. The mean ± SEM was used to establish the results (n = 6). A one-way ANOVA test was used, followed by Tukey’s *post hoc* test, and the represented *p*-value is <0.05.

Figure 3 demonstrates that compared to those in the control group, the cerebral levels of DA, NE, 5-HT, and GABA in the PA group were significantly lowered by 67.9%, 44.5%, 60.7%, and 73%, respectively. However, compared to that in the PA group, administration of *L. rhamnosus* resulted in a significant increase in DA, NE, 5-HT, and GABA levels by 1.5, 1.2, 1.6, and 1.4 times, respectively. Furthermore, compared to those in the PA group, Thy significantly increased the DA, NE, 5-HT, and GABA levels by 1.9, 1.3, 1.9, and 1.4 times, respectively. Co-administration of *L. rhamnosus* and Thy showed significantly greater increases in cerebral DA, NE, 5-HT, and GABA levels by 2.2-fold, 1.6-fold, 2.2-fold, and 1.5-fold, respectively.

### 3.3 Therapeutic effects of *L. rhamnosus*, Thy and their combination on the cerebral levels of neuroinflammatory biomarkers in PA-induced neurotoxicity

**FIGURE 4:**
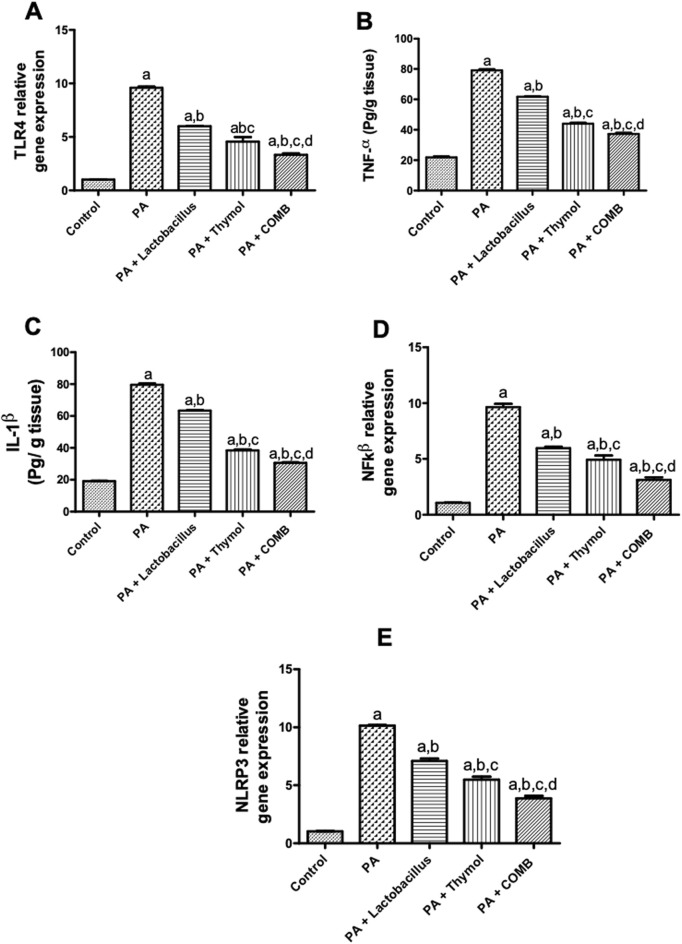
Therapeutic effects of *L. rhamnosus*, Thy and their combination on the cerebral levels of neuroinflammatory biomarkers in PA-induced neurotoxicity. **(A)** TLR-4, **(B)** TNF-α, **(C)** IL-1β, **(D)** NF-κB, and **(E)** NLRP3. **(a)** Significance relative to the control group. **(b)** Significance relative to the PA group. **(c)** Significance relative to the PA + *Lactobacillus rhamnosus* group. **(d)** Significance relative to the PA + Thy group. Significance: *p* < 0.05. PA, propionic acid. TLR-4, Toll-like receptor-4; TNF-α, tumor necrosis factor alpha; IL-1β, interleukin-1β; NF-κB: nuclear factor kappa B; NLRP3, nucleotide-binding domain, leucine-rich-containing family, pyrin domain-containing-3. The mean ± SEM was used to establish the results (n = 6). One way ANOVA test was used, followed by Tukey’s *post hoc* test, and the represented *p*-value is <0.05.

As shown in Figure 4, compared to that in the control group, PA significantly elevated the cerebral inflammatory biomarkers TLR4, TNF-α, IL-1β, NF-κβ, and NLRP3 by 9.4, 3.6, 4.2, 9.6, and 9.8 times, respectively. On the other hand, treatment with *L. rhamnosus* led to a significant decrease in the cerebral levels of TLR4, TNF-α, IL-1β, NF-κβ, and NLRP3 by 37.5%, 21.9%, 20.4%, 38.2%, and 29.7%, respectively, compared to that in the PA group. In parallel, compared to that in the PA group, administration of Thy reduced the cerebral levels of TLR4, TNF-α, IL-1β, NF-κβ, and NLRP3 by 52.5%, 44.3%, 51.8%, 49.2%, and 46%, respectively. Remarkably, compared to that in the untreated PA group, co-administration of *L. rhamnosus* and Thy had the highest inhibitory effects on the cerebral levels of TLR4, TNF-α, IL-1β, NF-κβ, and NLRP3 by 65.3%, 52.9%, 61.6%, 67.5%, and 61.9%, respectively (Figure 4).

### 3.4 Therapeutic effects of *L. rhamnosus*, Thy and their combination on the cerebral levels of oxidative stress biomarkers in PA-induced neurotoxicity

**FIGURE 5:**
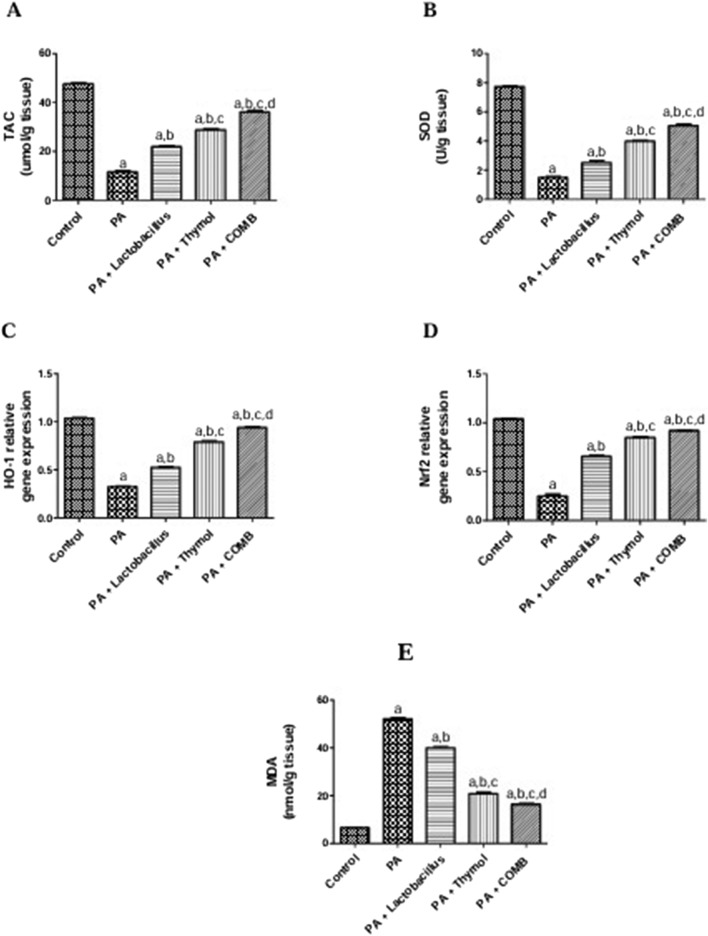
Therapeutic effects of *L. rhamnosus*, Thy and their combination on the cerebral levels of oxidative stress biomarkers in PA-induced neurotoxicity. **(A)** TAC, **(B)** SOD, **(C)** HO-1, **(D)** Nrf2, and **(E)** MDA. **(a)** Significance relative to the control group. **(b)** Significance relative to the PA group. **(c)** Significance relative to the PA + *Lactobacillus rhamnosus* group. **(d)** Significance relative to the PA + Thy group. Significance: *p* < 0.05. PA, propionic acid. TAC, total antioxidant capacity; SOD, superoxide dismutase; HO-1, heme oxygenase-1; Nrf2, nuclear factor erythroid 2-related factor 2; MDA, malondialdehyde. The mean ± SEM was used to establish the results (n = 6). A one-way ANOVA test was used, followed by Tukey’s *post hoc* test, and the represented *p*-value is <0.05.

As shown in Figure 5, administration of PA significantly increased the cerebral level of MDA in the brain by 7.9 times and drastically reduced the amounts of antioxidants TAC, SOD, *HO-1,* and *Nrf2* by 75.9%, 80.9%, 69%, and 75.9%, respectively, compared to that in the control group. Compared to that in the PA group, treatment with *L. rhamnosus* resulted in a 23.4% decrease in the content of MDA in the brain but a 1.9-, 1.7-, 1.6-, and 2.6-fold increase in TAC, SOD, *HO-1,* and *Nrf2* levels, respectively. In the same way, compared to that in the PA group, treatment with Thy showed a significant 60% decrease in the content of MDA in the brain with a 2.2-, 2.7-, 3-, and 3.4-fold increase in TAC, SOD, *HO-1,* and *Nrf2*, respectively. Co-administration of *L. rhamnosus* and Thy significantly led to a notable decrease in the content of MDA in the brain (68.6%) and an increase in TAC (3.1-fold), SOD (3.4-fold), HO-1 (2.9-fold), and Nrf2 (3.7-fold) levels compared to those in the PA group.

### 3.5 Therapeutic effects of *L. rhamnosus*, Thy and their combination on the cerebral levels of apoptotic biomarkers in PA-induced neurotoxicity

**FIGURE 6:**
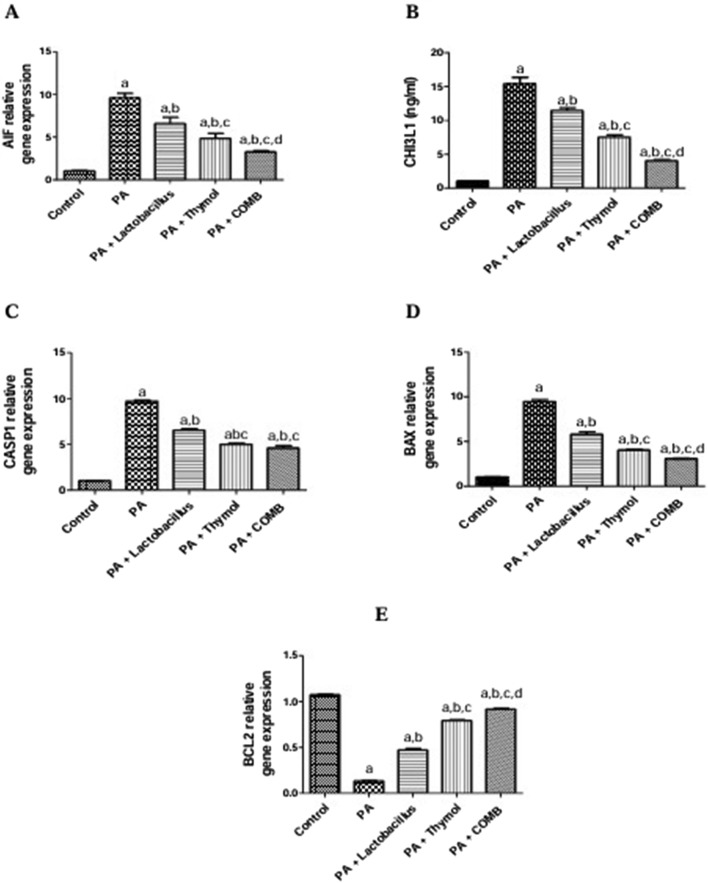
Therapeutic effects of *L. rhamnosus*, Thy and their combination on the cerebral levels of apoptotic biomarkers in PA-induced neurotoxicity. **(A)** AIF, **(B)** CHI3L, **(C)** CASP-1, **(D)** BAX, and **(E)** Bcl2. **(a)** Significance relative to the control group. **(b)** Significance relative to the PA group. **(c)** Significance relative to the PA + *Lactobacillus rhamnosus* group. **(d)** Significance relative to the PA + Thy group. Significance: *p* < 0.05. PA, propionic acid. AIF, apoptosis-inducing factor; CHI3L: chitinase-3-like protein 1; BAX: Bcl-2-like protein 4; Bcl2: B-cell lymphoma 2. The mean ± SEM was used to establish the results (n = 6). A one-way ANOVA test was used, followed by Tukey’s *post hoc* test, and the represented *p*-value is <0.05.

Treatment with PA increased the cerebral apoptotic biomarkers AIF, CHI3L, *caspase-1,* and *BAX* levels by 9.2, 14.9, 9.7, and 9.5 times, respectively, and decreased anti-apoptotic cerebral *Bcl2* levels by 87.6% compared to those in the control group (Figure 6). On the other hand, compared to that in the PA group, treatment with *L. rhamnosus* resulted in reversed apoptosis by increasing cerebral *Bcl2* levels by 3.5 times and decreasing the cerebral levels of *AIF*, CHI3L, *caspase-1*, and *BAX* by 31.2%, 25.8%, 32.6%, and 38.8%, respectively. Additionally, treatment with Thy increased the gene expression levels of *Bcl2* by 5.9 times compared to those in the PA group and inhibited the elevated cerebral levels of *AIF*, CHI3L, *caspase-1*, and *BAX* by 49.3%, 51.1%, 57.4%, and 26.2%, respectively, thereby mitigating apoptosis. Additionally, co-administration of *L. rhamnosus* and Thy led to a significant reduction in the cerebral levels of *AIF*, CHI3L, *caspase-1*, and *BAX* by 73.8%, 51.1%, and 67.7%, respectively, and significantly restored Bcl2 levels by 6.9 times compared to those in the PA group.

### 3.6 Therapeutic effects of *L. rhamnosus*, Thy and their combination on the cerebral *Wnt3/β-catenin/GSK3β* pathways in PA-induced neurotoxicity

**FIGURE 7:**
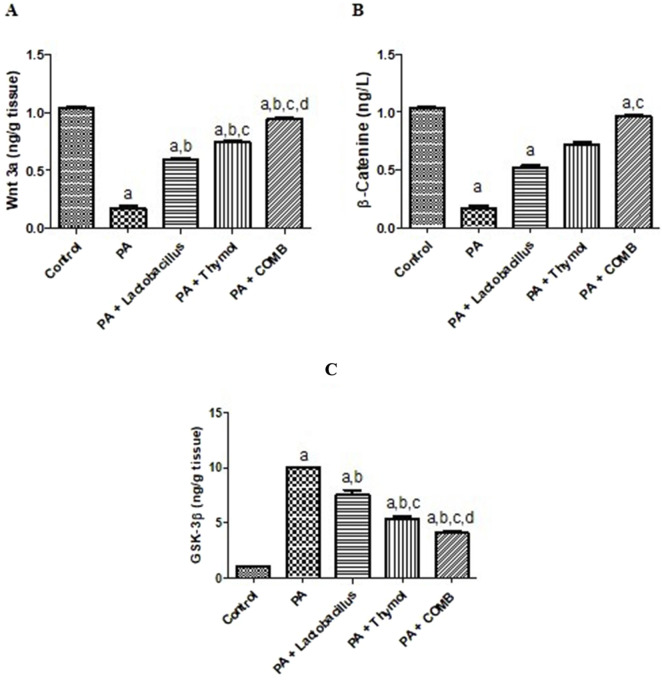
Therapeutic effects of *L. rhamnosus*, Thy and their combination on the cerebral Wnt3/β-Catenin/GSK3β pathways in PA-induced neurotoxicity. **(A)** Wnt3, **(B)** β-catenin, and **(C)** GSK3β in autistic rats. **(a)** Significance relative to the control group. **(b)** Significance relative to the PA group. **(c)** Significance relative to the PA + *Lactobacillus rhamnosus* group. **(d)** Significance relative to the PA + Thy group. Significance: *p* < 0.05. PA, propionic acid; GSK3β, glycogen synthase kinase-3β. The mean ± SEM was used to establish the results (n = 6). A one-way ANOVA test was used, followed by Tukey’s *post hoc* test, and the represented *p*-value is <0.05.

Treatment with PA reduced cerebral *Wnt3* and *β-catenin* levels significantly (83.7% and 51.4%, respectively) compared to those in the control group (Figure 7). At the same time, *GSK3β* gene expression was significantly upregulated by 9.9 times. However, these effects were significantly reversed upon treatment with *L. rhamnosus*, Thy and their combination compared to those in the PA group. Treatment with *L. rhamnosus* decreased the cerebral *GSK3β* gene expression levels by 23.4% and upregulated cerebral *Wnt3* and *β-catenin* levels by 3.4 and 3.1 times, respectively, compared to those in the PA group. Additionally, Thy decreased the expression of *GSK3β* levels by 45.9% while increasing the levels of *Wnt3* and *β-catenin* by 4.4 and 4.2 times, respectively, compared to that in the PA group. Notably, co-administration of *L. rhamnosus* and Thy produced the greatest ameliorative effect in restoring the *Wnt3/β-catenin/GSK3β* pathway compared with the PA group. This was demonstrated by a 5.5-fold increase in the *Wnt3* level, a 5.7-fold increase in the β-catenin level, and a 59.1% downregulation of the *GSK3β* expression levels compared to those in the PA group.

### 3.7 Therapeutic effects of *L. rhamnosus*, Thy and their combination on the cerebral ER stress and autophagy biomarkers in PA-induced neurotoxicity

**FIGURE 8:**
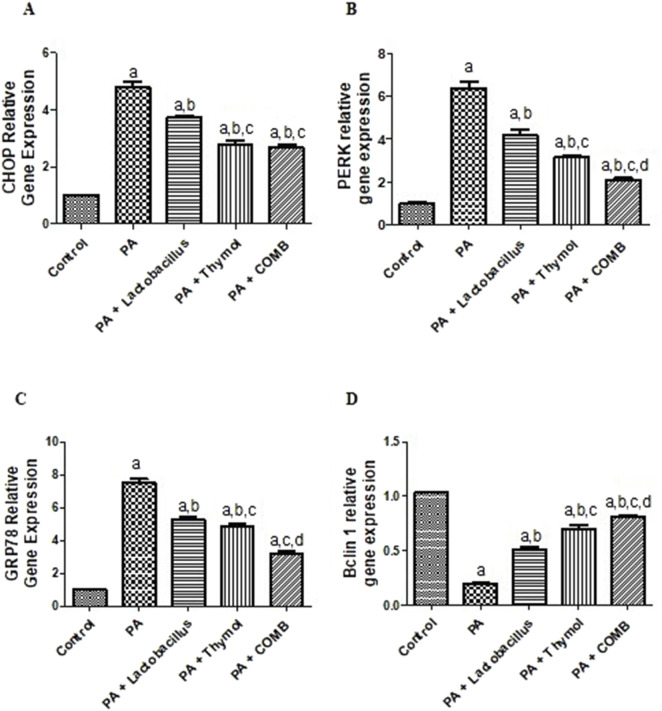
Therapeutic effects of *L. rhamnosus*, Thy and their combination on the cerebral ER stress and autophagy biomarkers in PA-induced neurotoxicity. **(A)** *CHOP*, **(B)** *PERK*, **(C)** *GRP78*, and **(D)** Beclin1. **(a)** Significance relative to the control group. **(b)** Significance relative to the PA group. **(c)** Significance relative to the PA + *Lactobacillus rhamnosus* group. **(d)**: Significance relative to the PA + Thy group. Significance: *p* < 0.05. PA, propionic acid; CHOP, C/EBP homologous protein; PERK, PKR-like ER kinase; GRP78, glucose regulated protein 78. The mean ± SEM was used to establish the results (n = 6). A one-way ANOVA test was used, followed by Tukey’s *post hoc* test, and the represented *p*-value <is 0.05.

As shown in Figure 8, compared to the control group, the PA group exhibited a significant downregulation of cerebral *Beclin1* gene expression by 5.3 times and a considerable elevation of cerebral *CHOP*, *PERK*, and *GRP78* gene expression levels (4.8-, 6.3-, and 7.5-fold, respectively). Conversely, compared to that in the PA group, treatment with *L. rhamnosus* showed a significant elevation of *Beclin1* gene expression by 2.5 times and a significant downregulation of *CHOP*, *PERK*, and *GRP78* gene expression by 23%, 33.3%, and 29.3%, respectively. In addition, compared to that in the PA group, Thy therapy significantly increased *Beclin1* gene expression by 3.5 times while considerably decreasing CHOP, PERK, and GRP78 gene expression levels by 41.7%, 51%, and 36%, respectively. By significantly reducing *CHOP*, *PERK*, and *GRP78* gene expression by 44%, 66.7%, and 57.3%, respectively, and increasing *Beclin1* gene expression by 4 times, the therapy with both *L. rhamnosus* and Thy in combination significantly had the greatest impact on the elevation of cerebral *Beclin1* gene expression and the lowest cerebral levels of CHOP, *PERK*, and *GRP78* gene expression levels compared to that in the PA group.

### 3.8 Therapeutic effects of *L. rhamnosus*, Thy and their combination on the cerebral BDNF/*p-TrkB*/CREB pathways in PA-induced neurotoxicity

**FIGURE 9:**
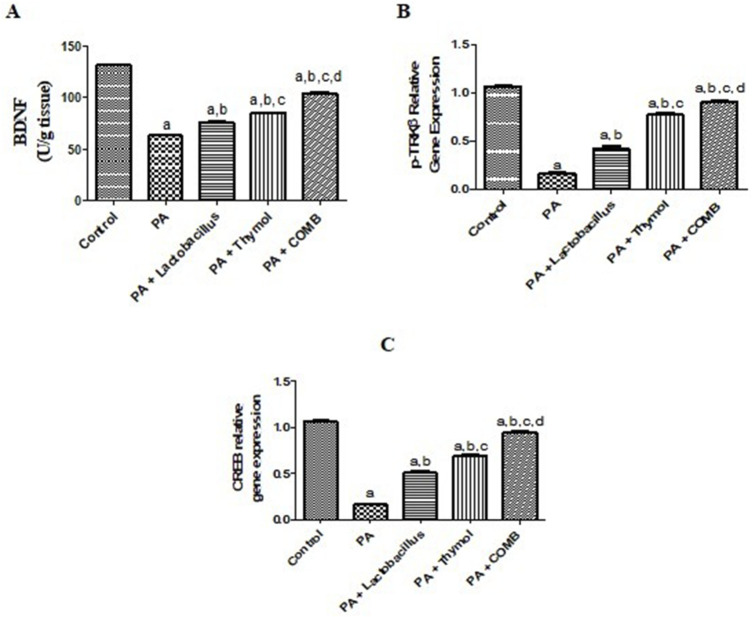
Therapeutic effects of *L. rhamnosus*, Thy and their combination on the cerebral BDNF/p-TrkB/CREB in PA-induced neurotoxicity. **(A)** BDNF, **(B)** p-TrkB, and **(C)** CREB. **(a)** Significance relative to the control group. **(b)** Significance relative to the PA group. **(c)** Significance relative to the PA + *Lactobacillus rhamnosus* group. **(d)** Significance relative to the PA + Thy group. Significance: *p* < 0.05. PA, propionic acid; BDNF, brain-derived neurotrophic factor; p-TrkB, phosphorylated tyrosine protein kinase; CREB, cAMP response element-binding protein. The mean ± SEM was used to establish the results (n = 6). A one-way ANOVA test was used, followed by Tukey’s *post hoc* test, and the represented *p*-value is <0.05.

Compared to that in the control group, PA significantly reduced the cerebral levels of BDNF, p-TrkB, and CREB by 52%, 82%, and 85%, respectively. Conversely, compared to that in the PA group, treatment with *L. rhamnosus* showed a significant increase in BDNF, p-TrkB, and CREB levels by 1.2-, 2.6-, and 3.2-fold, respectively. In addition, treatment with Thy showed a significant increase in the BDNF, p-TrkB, and CREB levels by 1.3-, 4.8-, and 4.3-fold, respectively. Interestingly, compared to that in the PA group, combined therapy with both *L. rhamnosus* and Thy significantly boosted BDNF, *p-TrkB*, and CREB levels by 1.7-, 5.7-, and 5.9-fold, respectively, showing the greatest improvement benefits for each individual therapy (Figure 9).

### 3.9 Therapeutic effects of *L. rhamnosus*, Thy and their combination on the cerebral pI3K/Akt/mTOR pathway in PA-induced neurotoxicity

**FIGURE 10:**
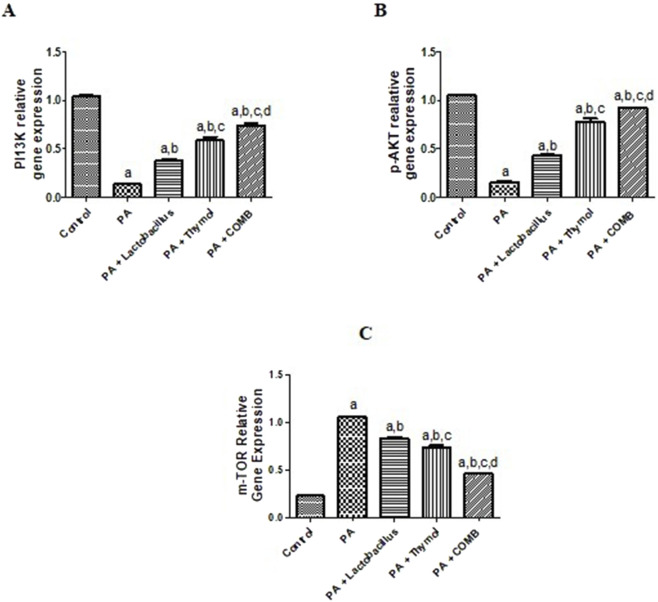
Therapeutic effects of *L. rhamnosus*, Thy and their combination on the cerebral pI3K/Akt/mTOR pathway in PA-induced neurotoxicity. **(A)** pI3K, **(B)** p-AKT, and **(C)** mTOR. **(a)** Significance relative to the control group. **(b)** Significance relative to the PA group. **(c)** Significance relative to the PA + *Lactobacillus rhamnosus* group. **(d)** Significance relative to the PA + Thy group. Significance: *p* < 0.05. PA, propionic acid; pI3K, phosphoinositide-3-kinase-Akt-mammalian; AKT, protein kinase B; mTOR, mechanistic target of rapamycin. The mean ± SEM was used to establish the results (n = 6). A one-way ANOVA test was used, followed by Tukey’s *post hoc* test, and the represented *p*-value is <0.05.

As shown in Figure 10, compared to the control group, the PA group exhibited a significant elevation of cerebral *mTOR* gene expression by 5-fold along with significant downregulation of cerebral pI3K and *Akt* gene expression by 7.6- and 6.7-fold, respectively (Figure 10). Conversely, compared to that in the PA group, administration of *L. rhamnosus* alone showed a significant downregulation of *mTOR* gene expression by 1.3 times and a significant elevation of *PI3K* and *Akt* gene expression by 3.1 and 2 times, respectively. Similarly, compared to that in the PA group, administration of Thy significantly decreased *mTOR* gene expression by 1.4 times while significantly increasing *PI3K* and *Akt* gene expression by 4.6 and 4 times, respectively.

### 3.10 Therapeutic effects of *L. rhamnosus*, Thy and their combination on the cerebral AMPK and SIRT1 pathways in PA-induced neurotoxicity

**FIGURE 11:**
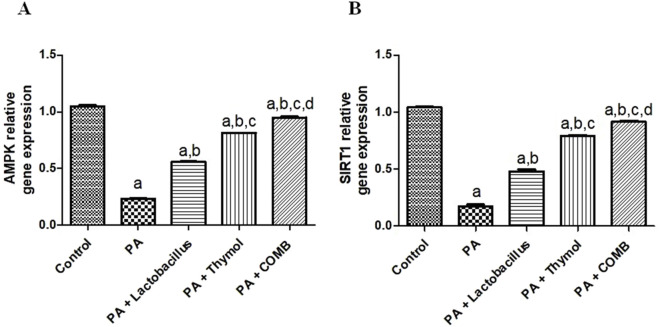
Therapeutic effects of *L. rhamnosus*, Thy and their combination on the cerebral AMPK and SIRT1 pathways in PA-induced neurotoxicity. **(A)** AMPK and **(B)** SIRT1. **(a)** Significance relative to the control group. **(b)** Significance relative to the PA group. **(c)** Significance relative to the PA + *Lactobacillus* rhamnosus group. **(d)** Significance relative to the PA + Thy group. Significance: *p* < 0.05. PA, propionic acid; AMPK, AMP-activated protein kinase; SIRT1, sirtuin 1. The mean ± SEM was used to establish the results (n = 6). One way ANOVA test was used, followed by Tukey’s *post hoc* test, and the represented *p*-value is <0.05.

As shown in Figure 11, cerebral *AMPK* and *SIRT1* levels were significantly reduced more in the PA group than in the control group by 4.2 and 5.7 times, respectively. However, compared to that in the PA group, these effects were significantly reversed after treatment with *L. rhamnosus*, Thy and their combination (p < 0.05). Compared that in to the PA group, treatment with *L. rhamnosus* increased the expression of the *SIRT1* and *AMPK* genes by 2.8 and 2.4 times, respectively. In addition, compared to that in the PA group, Thy elevated cerebral *SIRT1* and *AMPK* gene expression levels by 4.5 and 3.5 times, respectively. Notably, co-administration of both *L. rhamnosus* and Thy upregulated *AMPK* and *SIRT1* by 4 and 5.2 times, respectively, compared to that in the PA group. Interestingly, the combination of *L. rhamnosus* and Thy therapy exhibited the greatest ameliorating activity for the restoration of *AMPK* and *SIRT1* compared to the effects of solo therapy.

### 3.11 Therapeutic effects of *L. rhamnosus*, Thy and their combination on the cerebral histopathological changes in PA-induced neurotoxicity

**FIGURE 12:**
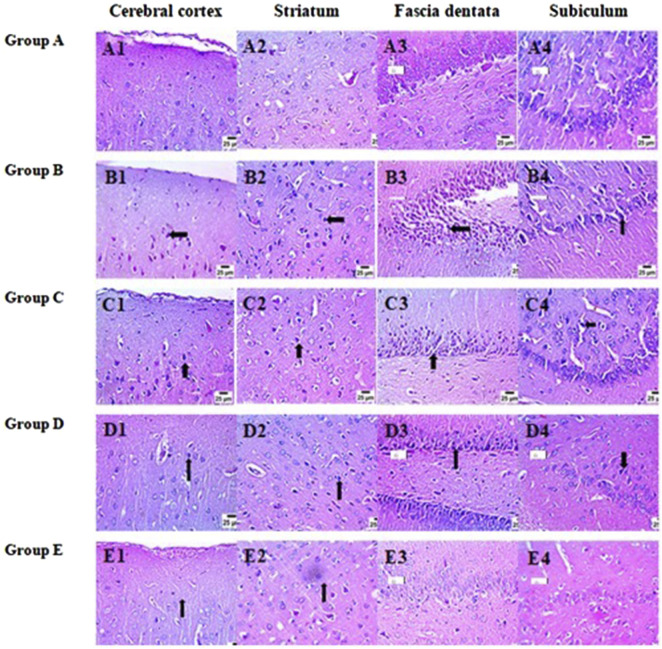
Therapeutic effects of *L. rhamnosus*, Thy and their combination on the cerebral histopathological changes in PA-induced neurotoxicity. (Group **(A)**) Control, (group **(B)**) PA, (group **(C)**) PA + *L. rhamnosus*, (group **(D)**) PA + Thy, and (group **(E)**) PA + *L. rhamnosus* + Thy. Nuclear pyknosis and abnormalities in brain sections are indicated by black arrows (scale bar 25 µm) (n = 4).

Histopathological evaluation of brain tissues was performed in our research; the photomicrographic results are provided in Figure 12.

In the control group, the photomicrographs showed no marked pathological changes in the cerebral cortex, striatum, fascia dentata, and subiculum (Figures 12A1, 4). However, the PA group showed a high number of necrotic neurons in the cerebral cortex (black arrow) (Figure 12B1), a high number of shrunken and degenerated neurons in the striatum (black arrow) (Figure 12B2), severe nuclear pyknosis in a high number of neurons in the fascia dentate (black arrow) (Figure 12B3), and nuclear pyknosis in a moderate number of neurons in the subiculum (black arrow) (Figure 12B4).

In contrast, upon treatment with *L. rhamnosus*, we detected a moderate number of shrunken and degenerated neurons (black arrow) in the cerebral cortex and striatum (Figures 12C1, 2), nuclear pyknosis (black arrow) in a moderate number of neurons in the fascia dentata (Figure 12C3), and nuclear pyknosis (black arrow) in a moderate number of neurons in the subiculum (Figure 12C4).

In the same way, the Thy group showed a few degenerated neurons in the cerebral cortex (black arrow; Figure 12D1), a moderate number of shrunken and degenerated neurons in the striatum (black arrow; Figure 12D2), nuclear pyknosis in a moderate number of neurons in the fascia dentata (black arrow; Figure 12D3), and nuclear pyknosis in a few neurons in the subiculum (black arrow; Figure 12D4).

The combined treatment group showed the highest therapeutic effect on brain tissues, where few numbers of degenerated neurons were observed in the cerebral cortex (black arrow; Figure 12E1), few numbers of necrotic neurons were observed in neurons in the striatum (black arrow; Figure 12E2), and no marked pathological changes were observed in the fascia dentata and subiculum (Figures 12E3, 4).

### 3.12 Therapeutic effects of *L. rhamnosus*, Thy and their combination on the immunohistochemical changes in the cerebral cortex in PA-induced neurotoxicity

**FIGURE 13:**
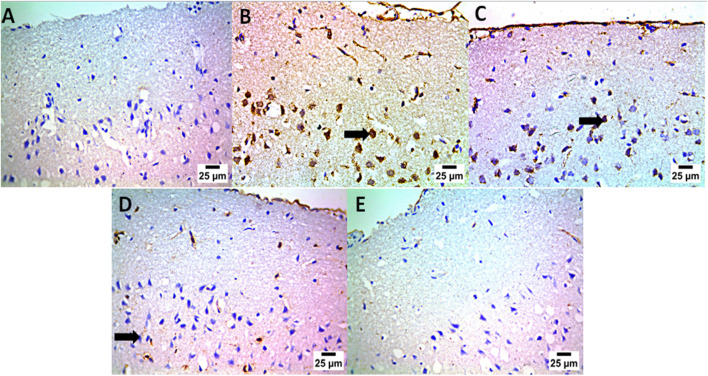
Effect of *L. rhamnosus*, Thy and their combination on the brain immunohistochemical changes in PA-induced neurotoxicity. (Group **(A)**) Control, (group **(B)**) PA, (group **(C)**) PA + *Lactobacillus*, (group **(D)**) PA + Thy, and (group **(E)**) PA + COMB. Abnormalities in the brain are indicated by black arrows (n = 4).

**FIGURE 14:**
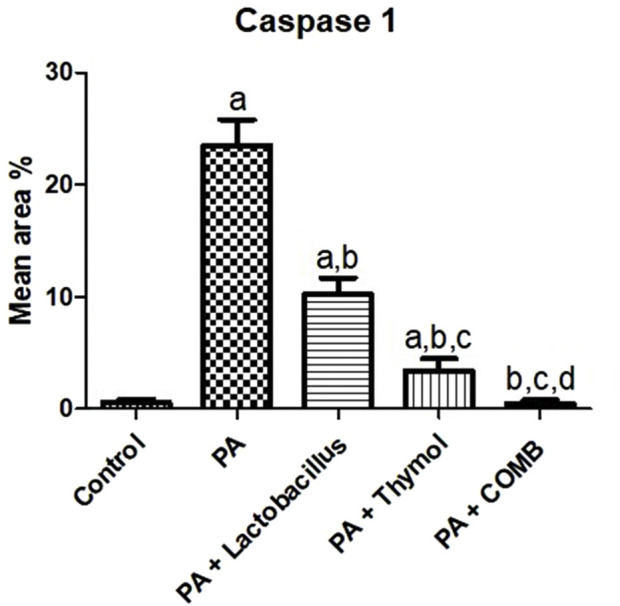
Quantitative analysis of the therapeutic effects of *Lactobacillus rhamnosus* (*L. rhamnosus*) and/or thymol (Thy) on the immunohistochemical changes in caspase-1 in the cerebral cortex in PA-induced neurotoxicity. (Group A) Control, (group B) PA, (group C) PA + *L. rhamnosus*, (group D) PA + Thy, and (group E) PA + *L. rhamnosus* + Thy. Abnormalities in the brain are indicated by black arrows. **(a)** Significance relative to the control group. **(b)** Significance relative to the PA group. **(c)** Significance relative to the PA + *Lactobacillus rhamnosus*. **(d)** Significance relative to the PA + Thy group. PA, propionic acid. The mean ± SEM was used to establish the results (n = 4). A one-way ANOVA test was used, followed by Tukey’s *post hoc* test, and the represented *p*-value is <0.05.

Immunohistochemical analysis of brain tissues was performed in our research, as illustrated in the photomicrographic results (Figure 13); groups A and E showed negative expression for caspase-1 in neurons of the cerebral cortex (the control group and PA + *L. rhamnosus* + Thy group). Upon using PA, high positive expression of caspase-1 occurred in the neurons of the cerebral cortex (group B), and moderate positive expression for caspase-1 was observed in the neurons of the cerebral cortex (group C). In group (D), mild positive expression of caspase-1 in the neurons of the cerebral cortex was detected. Statistical analysis in Figure 14 shows the reaction area percent of caspase-1, and the data are represented as the mean ± SD (n = 7); values indicate that these means of the group (PA + *L. rhamnosus* + Thy) were significantly more variable than that of the other group (*p* ≤ 0.0001) according to one-way ANOVA and Tukey’s tests.

### 3.13 Therapeutic effects of *Lactobacillus rhamnosus*, Thy and their combination on the immunohistochemical changes in the cerebral striatum in PA-induced neurotoxicity

**FIGURE 15:**
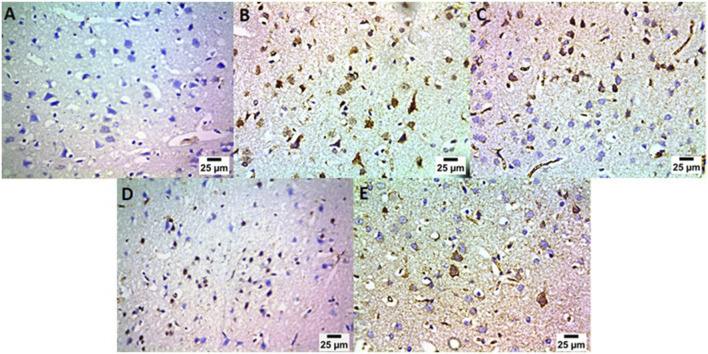
Therapeutic effects of *Lactobacillus rhamnosus* (*L. rhamnosus*) and/or thymol (Thy) on the immunohistochemical changes in the cerebral striatum in PA-induced neurotoxicity. (Group **(A)**) Control, (group **(B)**) PA, (group **(C)**) PA + *Lactobacillus*, (group **(D)**) PA + Thy, and (group **(E)**) PA + COMB. Abnormalities in the brain are indicated by black arrows (n = 4).

**FIGURE 16:**
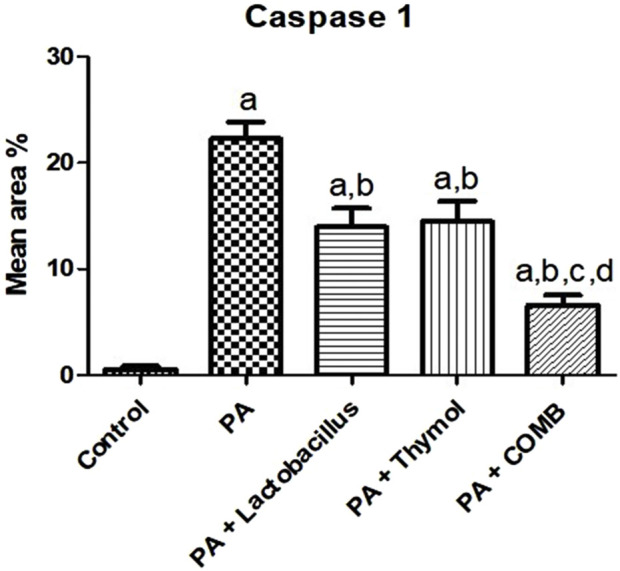
Quantitative analysis of the therapeutic effects of *Lactobacillus rhamnosus* (*L. rhamnosus*) and/or thymol (Thy) on the immunohistochemical changes of caspase-1 in the cerebral striatum in PA-induced neurotoxicity. (Group A) Control, (group B) PA, (group C) PA + L. rhamnosus, (group D) PA + Thy, and (group E) PA + *L. rhamnosus* + Thy. Abnormalities in the brain are indicated by black arrows. Data are presented as the means ± SE (n = 7). **(a)** Significance relative to the control group. **(b)** Significance relative to the PA group. **(c)** Significance relative to the PA + *Lactobacillus rhamnosus* group. **(d)** Significance relative to the PA + Thy group. PA, propionic acid. The mean ± SEM was used to establish the results (n = 4). A one-way ANOVA test was used, followed by Tukey’s *post hoc* test, and the represented *p*-value is <0.05.

The effect of treatment with *L. rhamnosus*, Thy and their combination on caspase-1 in the neurons of the striatum was studied in our research (Figure 15). Group (A) showed negative expression for caspase-1 in the neurons of the striatum in the normal control group. In group (B), a high positive expression for caspase-1 was detected in the neurons of the striatum in the control positive group. Regarding groups C and D, moderate positive expression for caspase-1 was found in the neurons of the striatum. In group (E), we detected mild positive expression for caspase-1 in the neurons of the striatum. Figure 16 showed the quantitatively estimated reaction area percentage of caspase-1, and data are represented as the mean ± SD (n = 7). Values indicate that the mean of group E was significantly more variable than that of other groups (p ≤ 0.0001) according to one-way ANOVA and Tukey’s tests.

## 4 Discussion

Our study investigated the neuro-therapeutic effects of *L. rhamnosus*, Thy and their combination on the histological features, behavioral outcomes, neurotransmitters, oxidative stress, ER stress, autophagy, inflammation, apoptosis, and neuronal integrity in a PA-induced ASD rat model (graphical abstract).

According to our findings, *L. rhamnosus*, Thy and their combination greatly improved social and cognitive behaviors, decreased oxidative damage, decreased inflammation, maintained neuronal integrity, and restored normal pathways (Xiong et al., 2023). To the best of our knowledge, this is the first research that studies how *L. rhamnosus*, Thy and their combination can be used to treat rats with PA-induced autism.

The Y-maze, CAR, and OFT tests are frequently used to analyze learning, memory, and exploratory anxious cognitive and deficit behavior (Salehi et al., 2018). Motor development was measured by evaluating the swimming performance. Swimming activity was lower in the PA group (Capibaribe et al., 2019). According to our findings, PA greatly reduced SAP, significantly prolonged latency, and significantly decreased rearing and the frequency of ambulation. PA increased the immobility score while drastically decreasing the swimming score. On the first and second days, the PA group showed an increased number of avoidance responses to the electric shock. Therefore, in line with earlier research, our findings demonstrated that higher PA exposure causes autism-like behavior in rats (Sunand et al., 2020; Bagcioglu et al., 2023). Similarly, several studies reported that PA causes repetitive motions, aberrant motor behavior, and social behavior impairment (Choi et al., 2018; Thomas et al., 2012).

Confirming our findings, we also noted that several behavior tests were conducted over the past 15 years to assess the characteristics of the PA model. Social impairment in the tested rats, both with strangers and with each other, was the most unexpected observation (Kılıç et al., 2025; Mirza and Sharma, 2018; Shams et al., 2019; MacFabe et al., 2007). Movement disorders were characterized by repeated, stereotyped behavior that lacks a suggested pattern. PA demonstrated impaired cognition (Ma et al., 2011; Shams et al., 2019; Ali et al., 2020), location avoidance (Eissa et al., 2020; Ossenkopp et al., 2012), repetitive behavior (Kamen et al., 2019; Shultz et al., 2009), rearing (Ali et al., 2020; Shultz et al., 2009), and hyposensitivity and reduced exploration (Kamen et al., 2019). It is well-known that PA, a metabolite typically produced by the overgrowth of bacteria such as clostridia and others, can effectively cause rodents to exhibit chronic autistic symptoms (Bin-Khattaf et al., 2022).

Our results showed that *L. rhamnosus*, Thy and their combination had therapeutic benefits on behavioral outcomes, with both medications significantly improving the results of the employed behavioral tests when taken either alone or in combination. In addition to showing a significant improvement in open-field test results, the therapeutic effect of *L. rhamnosus*, Thy and their combination may increase SAP%, with significant improvement in swimming scores and decreased immobility scores, associated with a significant reduction in the number of electric shock avoidance response trials, all of which suggested behavioral change. Thy was able to return the observed behavioral parameter to its typical values after co-treatment with both *L. rhamnosus* and Thy.

In accordance with our findings, tests on Lact1 showed that the treatment with *L. rhamnosus* strains improved the autistic-like behaviors. Additionally, lactobacilli were able to maintain motor performance (Capibaribe et al., 2019; Al-Salem et al., 2016) and the swimming rate. In addition, *L. rhamnosus* therapy restored spontaneous alteration and cognitive capacity in the maze test. Improved memory and navigation abilities are two ways in which *L. rhamnosus* therapy affects the gut–brain connection (Capibaribe et al., 2019). Likewise, it was discovered that *L. rhamnosus* treatment significantly enhanced autistic-like behaviors in mice and that after 3 weeks of treatment, social behavior impairments were reversed in this model. Therefore, *L. rhamnosus* treatment successfully reduced core ASD-like symptoms, indicating that it may be used therapeutically to improve core ASD symptoms over a longer treatment period (Rosenfeld, 2015).

Accordingly, earlier research showed that probiotic administration can considerably reduce ASD symptoms in both humans and animals (Park et al., 2025; Hsiao et al., 2013; West et al., 2013). Male mice were more inclined to interact with stranger mice after receiving *L. rhamnosus* treatment for 2 weeks in the model of ASD, indicating an improvement in sociability. Meanwhile, *L. rhamnosus* reduced freezing and self-grooming behaviors in female mice (Kałużna-Czaplińska et al., 2012).

Therefore, *L. rhamnosus* treatment successfully reduced core ASD-like symptoms, indicating that it may be used therapeutically to improve core ASD symptoms over a longer treatment period.

Regarding Thy, in accordance with our results, Thy’s antidepressant-like effects were favorably demonstrated by a decrease in immobility time in the forced swimming test. These tests are based on the premise that stressful events elicit fight-or-flight responses (Guo et al., 2022; Vasconcelos et al., 2015). Additionally, Cavalcanti et al. concluded that immobility is strongly linked to hopelessness and depressive-like behavior and that Thy was successful in reversing these characteristics (Salehi et al., 2018). In addition, Xiong et al. (2023) stated that in ASD rats, Thy therapy alleviates repetitive stereotyped behaviors, motor activity, and social impairments. Thy was administered to rats for a brief period of time to improve their social deficiencies (Xiong et al., 2023).

*In vitro* and *in vivo* studies have demonstrated that Thy can enhance gut microbiota (Steru et al., 1985). By producing neuroactive metabolites, the gut microbiota was shown to control rat behavior in an *in vivo* model (Liu et al., 2022). Thus, through the gut microbiome, Thy may influence ASD. Through proven behavioral models (hole-board and light/dark tests), several studies established Thy’s anxiolytic character, suggesting that its effects on OFT and FST may be obscured by decreased anxiety. The concurrent repair of biochemical and structural abnormalities in our investigation demonstrates that the advantages reflect a meaningful neurotherapeutic effect beyond simple sedation, even though this pharmacological action contributes to the observed behavioral improvement (Capibaribe et al., 2019). However, the simultaneous restoration of several biochemical pathways and neural integrity indicates that the advantages go beyond simple sedation.

Therefore, behavioral studies, biochemical indicators, and literature data demonstrated that the medication dose employed in our investigation was both safe and effective in improving PA-induced neurobehavioral deficits. This creates several opportunities for Thy’s clinical use. It is important to note that the behavioral tests used in this work (Y-maze, CAR, OFT, and FST) primarily evaluate depressive-like behavior, anxiety, and cognitive flexibility, which are common comorbidities in ASD. To directly evaluate fundamental social deficiencies, future research should include social interaction tests (such as the three-chamber exam).

In the pathophysiology of ASD, alterations of the monoaminergic neurotransmitter have been found in numerous brain areas and in the peripheral system (Sharon et al., 2019).

Our results showed that PA-induced autistic rats showed diminished levels of brain neurotransmitters DA, NE, 5-HT, and GABA. Our findings are consistent with those of other studies that showed that the decrease in brain monoamines may be the cause of the observed deterioration in the behavioral outcomes in the PA group (Kuo and Liu, 2022; Rahi et al., 2021). Moreover, according to earlier research, dopaminergic dysfunction, particularly DA imbalance in a particular brain region, may be the etiology of ASD (Tiwari et al., 2021). Neurodevelopmental disorders such as ASD are associated with changes in the distribution of serotonin and NE (Marotta et al., 2020; Kubota et al., 2020). Numerous studies have demonstrated that autism, particularly in the early stages of brain development, exhibits an imbalance in serotonin levels (Tiwari et al., 2021; Hwang et al., 2017), where lower 5-HT availability was observed in the brain stem and the total gray matter (Muller et al., 2016). Accordingly, glutamate and GABA were altered in the PA model, which was in contrast to the control (Andersson et al., 2021; MacFabe, 2013).

According to the majority of these studies, autism is associated with lower GABA levels (El-Ansary et al., 2011; Puts et al., 2017; Rojas et al., 2014). Other research, however, has shown that individuals with ASD have higher GABA levels. While Cid-Jofré et al. (2022) showed that the GABA level increased in their experimental autism study, Maier et al. (2022) and El-Ansary and Al-Ayadhi (2014) revealed that the GABA level was higher in individuals with autism.

Additionally, some research has clarified how GABA affects neuroinflammation (Crowley et al., 2016). GABA balance can be impacted by several processes, and neuroinflammation can significantly exacerbate an imbalance. More research is required to ascertain whether GABA neurotransmission is compromised in autistic cases and elucidate how modafinil affects GABA (Sunand et al., 2020).

Our findings demonstrated the anti-autistic effects of *L. rhamnosus*, Thy and their combination, as demonstrated by a significant increase in DA, NE, 5-HT, and GABA.

In accordance with our results, Wei et al. (2019) recorded that *L. rhamnosus* was also able to reverse the reduction in the serotonin and dopamine levels in mice. Similarly, Desbonnet et al. (2008) reported enhancement of the neurotransmitters NE, dopamine, and serotonin in probiotic-autistic rats.

Accordingly, it was recorded that probiotics can promote the availability of additional monoamine precursors, function as a digestive aid to improve nutritional contents, replace pathogenic microorganisms, and ultimately increase serotonin, dopamine, and NE levels. Additionally, probiotics increased the expression of synaptophysin mRNA and dopamine receptor subtypes D1 and D2, along with the cellular response and monoamine release (Rehman et al., 2022).

Furthermore, investigations provide compelling proof that *L. rhamnosus* has beneficial effects on dopamine function and metabolism (Hamamah et al., 2022). In addition, two crucial enzymes in dopamine metabolism, tyrosine hydroxylase and dopamine β-hydroxylase, along with plasma cortisol, were decreased by *Lactobacillus plantarum* DR7 (Liu et al., 2020).

Additionally, studies revealed that as GABA-producing organisms, *L. rhamnosus* may be strong candidates for altering the glutamate/GABA ratio, making them potentially effective treatments for social behavioral symptoms associated with ASD (Yunes et al., 2020; Mintál et al., 2022; El-Ans and ary, 2024). In line with earlier research, Thy increased DA and NE levels in the brain (Mirza and Sharma, 2018; Javed et al., 2019; Kassab and El-Hennamy, 2017; Kuzay et al., 2022; Ogaly et al., 2022; Abu-Elfotuh et al., 2022b; Esnafoglu et al., 2017; Khayyat et al., 2025; El-Ansary, 2016). Thy-pretreatment showed significant increases in catecholamine for DA, NE, and 5HT (Abu-Elfotuh et al., 2022b).

Neurological problems are directly linked to oxidative stress; it was found that administration of PA treatment affects oxidative stress markers (Shultz et al., 2009; Rahi et al., 2021). In light of this result, we concluded that the development of autism is significantly influenced by oxidative stress. Our results showed that in contrast to the control group, PA-treated rats in our study exhibited a substantial increase in the oxidative stress marker MDA and a decrease in the levels of the antioxidants TAC, SOD, HO-1, and Nrf2. In rats administered PA, the therapeutic effect of *L. rhamnosus*, Thy and their combination markedly increased the antioxidant levels and decreased oxidative stress indicators.

Overproduction of reactive oxygen species is linked to oxidative stress in macrophages and microglia, which starts a destructive cycle of neuroinflammation and cellular damage. The pathophysiology of autism spectrum diseases, which includes behavioral abnormalities, cell death, and neuronal malfunction, is significantly influenced by these processes (Kılıç et al., 2025).

These findings are in line with earlier research by Esnafoglu et al. (2017), Tiwari et al. (2021), and Khayyat et al. (2025), who found that oxidative stress is a major mechanism of PA toxicity. Our findings were also consistent with several earlier studies that showed changes in several biomarkers linked to glutamate excitotoxicity and oxidative stress in a rat model of autism produced by PA (Bin-Khattaf et al., 2022; El-Ansary, 2016; El-Ansary et al., 2012; Aldbass et al., 2013).

Similarly, other research showed elevated oxidative stress markers in ASD (Alfawaz et al., 2022; Erbas et al., 2018; Kamalmaz et al., 2023; MacFabe et al., 2008); the elevated MDA levels in the PPAS group validated the role of oxidative stress in the PA-induced autism model (Sunand et al., 2020). In accordance with our results, Hussein et al. (2023) investigated gated Thy’s ability to prevent SOD activity, and the measurement of MDA and GSH levels was used to assess the antioxidant activity. Moreover, Gago et al. (2025) claimed that Thy prevents oxidative stress.

In our work, PA modulated the TLR4, TNF-α, IL-1β, NF-κβ, and NLRP3 inflammasome pathways, indicating an inflammatory response. However, treating PA-induced autistic rats with *L. rhamnosus*, Thy and their combination dramatically lessened the negative effects of PA on these markers, suggesting that these medications have anti-inflammatory properties. Remarkably, the group treated with the combination of both agents showed superior outcomes compared with the MSG-treated group and the groups receiving either treatment alone.

Numerous investigations in agreement with our results have demonstrated a strong correlation between ASD and inflammatory cytokines (Xie et al., 2017). Abu-Elfotuh et al. (2022b) Kılıç et al. (2025) assessed the effects of PA administration on key indicators of inflammation, including TLR4, TNF-α, IL-1β, NF-κβ, and the NLRP3 inflammasome. Increased astrocyte or microglia activity has been shown to produce aberrant immunological profiles, which are implicated in the pathophysiology of ASD (Petrelli et al., 2016).

In addition, numerous studies have demonstrated that TLR4 activates the NF-κB pathway, which, in turn, causes the production of inflammatory mediators (Kumar, 2019; Sharif et al., 2007). The NF-κB pathway plays a role in the development and onset of inflammatory disorders by regulating the inflammatory subcellular events (Guo et al., 2022). Furthermore, the generation of IL-1β is triggered by the activation of the NLRP3 inflammasome on astrocytes, which binds to its receptors on glial cells and intensifies the inflammatory response.

In accordance with our results, research reported that *L. rhamnosus* showed anti-inflammatory properties (Rosenfeld, 2015; Guo et al., 2015; Martinon and Tschopp, 2007; Song et al., 2017). This could occur through interactions with the altered microbiota, leading to modulation of the serotonergic system by reducing gut permeability and modifying inflammatory processes (Corridoni et al., 2012; Leber et al., 2012; Lyte et al., 2020; O’Mahony et al., 2015; Ruiz et al., 2017).

Many studies agreed with our results and showed that Thy appears to be safe in terms of side effects and has been demonstrated to reduce inflammation by reducing the initiation and advancement of the inflammatory processes in many animal models of human diseases (Meeran et al., 2016; Meeran et al., 2015; Riella et al., 2012). Moreover, Javed et al. (2019) found that Thy administration dramatically decreased pro-inflammatory cytokine release and activation, as demonstrated by the decreased levels in the rats’ brain tissues.

Accordingly, it has been shown that Thy reduced inflammation via controlling the NF-κB pathways mediated by TLR4 (Wu et al., 2017). Additionally, Thy may exert its previously reported antidepressant effect by inhibiting the activation of the NLRP3 inflammasome, which, in turn, may reduce the production of caspase-1 (Zhang et al., 2021). Accordingly, in Zhao et al., Thy therapy significantly decreased the production of these pro-inflammatory factors. Inflammatory receptors such as TLR can be stimulated to activate NF-κB, which is well-known for controlling inflammation and immunity (Nennig and Schank, 2017).

According to these experimental results, Thy inhibited the phosphorylation of IκB, which, in turn, decreased NF-κB’s translocation to the nucleus and the subsequent release of inflammatory mediators triggered by NF-κB (Zhao et al., 2024). It has been discovered that neuronal apoptosis is linked to autistic behavior. A previous study established this association by demonstrating increased BAX expression and decreased BCL2 expression in the hippocampus tissues of a mouse model of autism (Ming et al., 2022). While extra *Bax* speeds up apoptotic cell death and *AIF* translocation causes apoptosis in a caspase-independent manner, BCL2 preserves the integrity of the mitochondrial membrane, favoring cell survival (Mathew and Keerikkattil, 2021).

Similarly, our results showed that reduced anti-apoptotic BCL2 and increased pro-apoptotic apoptotic factors, including *AIF*, CHI3L, *CASP1*, and *BAX*, indicated enhanced apoptosis in the PA group. On the other hand, treatment with *L. rhamnosus*, Thy and their combination reversed apoptosis by lowering the apoptotic factors and increasing the anti-apoptotic BCL2.

Our findings were consistent with a prior study that found that Thy treatment had anti-apoptotic effects, as evidenced by a significant increase in *Bcl-2* mRNA expression by 8.2- and 9-fold, respectively, and a significant decrease in *Bax* and *AIF* mRNA expression by a similar amount compared to that in the PA group (Xiong et al., 2023). In addition, according to Amin et al. (2023), *L. rhamnosus* can interact with proteins that control the inherent resistance to apoptosis. By activating pro-caspases, downregulating anti-apoptotic Bcl-2, and upregulating pro-apoptotic Bax proteins, lactobacilli can overcome this resistance (Amin et al., 2023; Wang et al., 2025).

Many studies agreed with our results regarding Thy as an apoptotic factor; by interfering with mitochondrial membrane potential and ROS production, Thy treatment shows a decrease in cell number and cell apoptosis (Thapa et al., 2019; Chauhan et al., 2017). De La Chapa et al. (2018) also observed that Thy reduced HeLa cell viability and PARP cleavage-induced death, indicating that mitochondrial malfunction and subsequent apoptosis are the cause of Thy’s action.

It is evident that long-term ER stress may play a role in the buildup of misfolded proteins, which leads to neuronal death and subsequent neurodegeneration (Mou et al., 2020). GRP78 activates PERK in ER stress, which activates CHOP, a pro-apoptotic factor. CHOP can cause a series of detrimental effects on neurons, including the production of ROS, inflammation, and apoptotic cascades, by decreasing the anti-apoptotic protein BCL-2 and increasing the pro-apoptotic proteins BAX, AIF, and CASP1 (Saha et al., 2021).

Our findings demonstrated that PA-induced autistic rats exhibited elevation of CHOP, PERK, and GRP78 gene expression, which are indicators of prolonged ER stress, in accordance with the aforementioned cascade. Surprisingly, when administering *L. rhamnosus*, Thy and their combination, these negative effects are reversed (Bouhtit et al., 2021). Our results were consistent with other research that demonstrated that Thy may have an effect by causing the expression of procaspase-3 and procaspase-9 to decrease while CHOP expression increases. *In vivo* and *in vitro*, ROS can cause ER stress or *vice versa*, and CHOP is implicated in reticular stress (Han et al., 2018).

Our results showed that compared to those in the control group, the PA group’s levels of BDNF, p-TrkB, CREB, AMPK, and SIRT1 were highly reduced. This was accompanied by a significant decrease in Wnt3 and β-catenin levels and a concurrent increase in GSK3β gene expression. In contrast to those in the PA group, these alterations were significantly reversed by treatment with *L. rhamnosus* and Thy. The observed modulation of a neurotrophic signaling cascade, which may be the cause of the enhanced cognitive function, is consistent with the reported increase in BDNF and CREB after *L. rhamnosus*, Thy and their combination therapy.

According to Abu-Elfotuh et al., Thy dramatically increased the content of BDNF in the brain, Wnt3a, and β-catenin mRNA expression, which is consistent with our findings (Xiong et al., 2023). In addition to activating the BDNF pathway, Thy was discovered to have anti-oxidative and anti-inflammatory properties that contribute to its neuro-therapeutic benefits (Yang et al., 2015). Similarly, GSK-3β activity is reduced by BDNF overexpression, but the major Wnt signaling molecules and their downstream target β-catenin are upregulated (Yang et al., 2015). Accordingly, β-catenin’s nuclear translocation and the transcription of its target genes, including BDNF, are caused by the stimulation of the Wnt/β-catenin pathway (Zhang et al., 2018; Yi et al., 2012). On the other hand, the observed neuro-therapeutic effects in midbrain dopaminergic neurons are caused by the inhibition of inflammation through the activation of the Wnt pathway (L’episcopo et al., 2011).

Interestingly, studies showed that Thy counteracted the downregulation of β-catenin and Wnt3a (Ojetunde, 2024). Thus, Wnt3/β-catenin activation upregulates BDNF, which is essential for synaptic plasticity and reversing learning and memory deficits (Mohamed et al., 2021; Abu-Elfotuh et al., 2022c).

Compared to the control group, our results demonstrated that PA-induced autistic rats showed a significant elevation of mTOR gene expression and a significant downregulation of pI3K and Akt gene expression. Conversely, pI3K and Akt gene expression were significantly upregulated following *L. rhamnosus*, Thy and their combination treatment.

Our results were consistent with prior research showing that mTOR inhibition can enhance social interaction and PI3K/AKT/mTOR-mediated autophagic activity in ASD, offering a new target and avenue for ASD treatment. According to Poultney et al. (2013), autophagy-related genes have exonic copy number variation mutations linked to ASD. This suggests that autophagy failure is a contributing factor to ASD. Through the IGF-1/PI3K/AKT/mTOR pathway, the mammalian target of rapamycin (mTOR), a master regulator of cell proliferation, cellular metabolism, and autophagy, has been implicated in the development of ASD (Bozdagi et al., 2013). In this context, we believe that autophagy may be promoted or suppressed through the PI3K/AKT/mTOR pathway, thereby influencing the development of ASD (Yang et al., 2015).

Regarding the impact of Thy on autophagy, Dou et al. (2022) found that Thy significantly restored autophagy by measuring the levels of genes and proteins associated with autophagy. This suggests that Thy suppresses the inflammasome by inducing autophagy. Furthermore, the mechanism by which Thy regulates autophagy via the AMPK/mTOR axis is a well-known and traditional route among the many routes that control autophagy (Li et al., 2022). Autophagy is triggered when AMPK is activated as p-AMPK as it inhibits the conversion of mTOR to p-mTOR (Yang et al., 2019). Our findings were consistent with these results.

Furthermore, according to studies, Thy administration significantly decreased the phosphorylation of PI3K, Akt, and mTOR. PI3K’s main downstream target is Akt, which is also referred to as protein kinase B (PKB) (Liu et al., 2020; Herrera-Bravo et al., 2024), and one of Akt’s downstream effectors is mTOR. A key regulator linked to cellular autophagy is the PI3K/Akt/mTOR pathway (Wang et al., 2020; Ye et al., 2020). It is worth mentioning that inflammation can be decreased by promoting microglial autophagy (Chen et al., 2024; Hegdekar et al., 2023).

It has previously been demonstrated that administering *L. rhamnosus* to elderly rats can improve memory and decrease mTOR activation in the hippocampus, suggesting that it is effective in reducing age-related degenerative dementia (Kumar et al., 2020; Jeong et al., 2015). According to another investigation, the mTOR and Wnt/β-catenin pathways can be differently modulated by the culture of *L. rhamnosus* (Taherian-Esfahani et al., 2016). Another study reported similar results in a mouse model (Fu et al., 2017). Furthermore, it has been demonstrated that *L. rhamnosus* activates apoptosis by suppressing the expression of NF-κB and lowering the phosphorylation of mTOR elements such as Akt, PI3K, and p70S6 kinase (Hwang et al., 2013).

Recently, Wang et al. (2025) confirmed that *L. rhamnosus* exopolysaccharides induce autophagy and apoptosis and reduced the expression of phosphorylation levels of PI3K, AKT, and mTOR.

Our results demonstrate that exopolysaccharides from *Lactobacillus plantarum* reduce the expression of the phosphorylation levels of PI3K, AKT, and mTOR and cause autophagy and apoptosis.

One of the suggested mechanisms for *L. rhamnosus* is via neuroactive metabolite synthesis, such as SCFAs like butyrate, although this effect was not particularly assessed in this study.

SCFAs promote the secretion of gastrointestinal peptides and glucagon-like peptide 1 from the intestinal L-cell, which directly or indirectly inhibit NLRP3 inflammasome activation and ROS (Jackson et al., 2019; Scuto et al., 2024).

SCFA supplementation effectively reversed neuropathological aspects such as apoptosis activation, neuroinflammation amplification, and synaptic density loss. Possible therapeutic approaches include microbiota-derived SCFAs that may exert neuroprotection through BDNF-dependent PI3K/Akt signaling (Liu et al., 2025). Moreover, SCFAs’ bioactive polyphenol metabolites have the ability to influence particular pathways that determine the modulation of particular target genes; specifically, the Nrf2 pathway is activated to produce its antioxidant action (Zhou et al., 2019; Cuciniello et al., 2023).

### 4.1 Limitations

It is worth mentioning that the acute nature of the PA model represents a limitation in our study. Therefore, future studies should investigate the effects of a chronic PA-induced autism model and compare outcomes between the acute and the chronic models.

It is crucial to remember that the PA model causes significant, pervasive neurochemical alterations that might indicate an acute neurotoxic condition. The results should be evaluated with the knowledge that this model may not accurately capture the more nuanced and developmental pathophysiology of human ASD, even though it helps screen therapeutic drugs.

It is important to clarify that the role of microbiota modulation as a suggested mechanism underlying the observed effects was hypothesized based on existing literature rather than being directly tested in this study.

The use of whole-brain homogenates for biochemical and molecular analyses represents a major limitation of the study. This method may obscure region-specific changes in nuclear circuits, including the prefrontal cortex, hippocampus, and cerebellum, which are crucially involved in ASD. Future research should concentrate on analyzing these particular brain areas.

While one-way ANOVA with Tukey’s test was used for all comparisons, the problem of multiple comparisons should be taken into account when interpreting the many important findings from the exploratory analyses.

## 5 Conclusion

The exposure to PA may lead to autistic behavior presented by abnormalities in neurotransmitter levels, oxidative stress, pro-inflammatory factor release, autophagy, ER stress, and apoptosis. PA-induced neurotoxicity and behavioral deficits were reduced by *L. rhamnosus* and Thy, with their combined therapy demonstrating stronger effects. The normalization of oxidative stress, inflammatory, apoptotic, and neurotrophic indicators was linked to these improvements. The results are still preliminary and model-specific, but they are encouraging. Microbiome analysis, region-specific molecular assays, route validation studies, and behavioral testing tailored to ASD should all be part of future research.

## Data Availability

The original contributions presented in the study are included in the article/Supplementary Material, further inquiries can be directed to the corresponding author/s.

## Glossary

5-HT: Serotonin
AIF: Apoptosis-inducing factor
AKT: Protein kinase B
AMPK: AMP-activated protein kinase
ASD: Autism spectrum disorder
BAX: Bcl-2-like protein 4
Bcl2: B-cell lymphoma 2
BDNF: Brain-derived neurotrophic factor
CAR: Conditioned avoidance response
CHI3L: Chitinase-3-like protein 1
CHOP: C/EBP homologous protein
CREB: Camp response element-binding protein.
DA: Dopamine
ELISA: Enzyme-linked immunosorbent assay
ER: Endoplasmic reticulum
FST: Forced swimming test
GABA: Gamma-aminobutyric acid
Glu: Glutamate
GRP78: Glucose regulated protein 78
GSK3β: Glycogen synthase binase-3 beta
GSK3β: Glycogen synthase kinase-3β
HO-1: Heme oxygenase-1
IDS: Intellectual disability
IL: Interleukin
L. rhamnosus: *Lactobacillus* rhamnosus
MDA: Malondialdehyde
mTOR: Mechanistic target of rapamycin
Nf-κb: Nuclear factor kappa B
NGS: Next-generation sequencing
NLRP3: Nucleotide-binding domain, leucine-rich-containing family, pyrin domain-containing-3
Nrf2: Nuclear factor erythroid 2-related factor 2
PA: Propionic acid
PERK: PKR-like ER kinase
PGE2: Pro-inflammatory cytokines like prostaglandin E2
PI3K: Phosphoinositide-3-kinase-Akt-mammalian
p-Trkb: Phosphorylated tyrosine protein kinase
QPCR: Quantitative real-time PCR
SAB: Spontaneous alternation behavior
SCFAS: Short-chain fatty acids
SIRT1: Sirtuin 1
SOD: Superoxide dismutase
TAC: Total antioxidant capacity
Thy: Thymol
TLR-4: Toll-like receptor-4
TNF: Tumor necrosis factor (TNF)-Α
TOC: Total antioxidant capacity
