# Supplementary material for: Therapeutic effects of Lactobacillus rhamnosus, thymol and their combination against neurotoxicity in propionic acid (PA)-induced autistic rats: insights into the role of the Nrf2/HO-1, Wnt3/β-catenin/GSK3β BDNF/p-TrkB/CREB, pI3K/Akt/mTOR, AMPK/SIRT-1, and PERK/CHOP/Bcl-2 pathways
Source: Front Pharmacol. 2026 Jan 28;16:1728908. doi: 10.3389/fphar.2025.1728908 (PMC12891102; doi:10.3389/fphar.2025.1728908)
Supplement: Supplementary file 1 [file Supplementaryfile1.docx]

Supplementary table1 statistical values of FST immobility

| FST immobility | Mean Diff. | q | Significa  nt? P < 0.05? | Summ ary | 95% CI of diff |
| --- | --- | --- | --- | --- | --- |
| Control vs PA | -40.00 | 82.61 | Yes | *** | -42.01 to -37.99 |
| Control vs PA +  Lactobacillus | -8.167 | 16.87 | Yes | *** | -10.18 to -6.155 |
| Control vs PA + Thymol | -2.500 | 5.163 | Yes | ** | -4.512 to -0.4880 |
| Control vs PA + COMB | -1.167 | 2.410 | No | ns | -3.179 to FST immobility  0.8453 |
| PA vs PA + Lactobacillus | 31.83 | 65.74 | Yes | *** | 29.82 to 33.85 |
| PA vs PA + Thymol | 37.50 | 77.45 | Yes | *** | 35.49 to 39.51 |
| PA vs PA + COMB | 38.83 | 80.20 | Yes | *** | 36.82 to 40.85 |
| PA + Lactobacillus vs PA + | 5.667 | 11.70 | Yes | *** | 3.655 to 7.679 |

| Thymol |  |  |  |  |  |
| --- | --- | --- | --- | --- | --- |
| PA + Lactobacillus vs PA +  COMB | 7.000 | 14.46 | Yes | *** | 4.988 to 9.012 |
| PA + Thymol vs PA +  COMB | 1.333 | 2.754 | No | ns | -0.6787 to 3.345 |

Supplementary table2 statistical values of FST swimming

| FST  swimming | Mean Diff. | q | Significant  P < 0.05? | Summary | 95% CI of diff |
| --- | --- | --- | --- | --- | --- |
| Control vs PA | 39.83 | 13.00 | Yes | *** | 27.10 to 52.57 |
| Control vs PA  +  Lactobacillus | 15.00 | 4.895 | Yes | * | 2.267 to 27.73 |
| Control vs PA  + Thymol | 14.67 | 4.786 | Yes | * | 1.933 to 27.40 |
| Control vs PA  + COMB | 8.833 | 2.883 | No | ns | -3.900 to 21.57 |
| PA vs PA +  Lactobacillus | -24.83 | 8.104 | Yes | *** | -37.57 to -12.10 |
| PA vs PA +  Thymol | -25.17 | 8.213 | Yes | *** | -37.90 to -12.43 |
| PA vs PA +  COMB | -31.00 | 10.12 | Yes | *** | -43.73 to -18.27 |
| PA +  Lactobacillus vs PA +  Thymol | -0.3333 | 0.1088 | No | ns | -13.07 to 12.40 |
| PA + | -6.167 | 2.012 | No | ns | -18.90 to 6.567 |

| Lactobacillus vs PA +  COMB |  |  |  |  |  |
| --- | --- | --- | --- | --- | --- |
| PA +  Thymol vs PA + COMB | -5.833 | 1.904 | No | ns | -18.57 to 6.900 |

Supplementary table3 statistical values of 1^st^ day condition

| Tukey's Multiple Comparison Test | | | | | |
| --- | --- | --- | --- | --- | --- |
| 1^st^ day  condition | Mean Diff. | q | Significant? P  < 0.05? | Summary | 95% CI of diff |
| Control vs PA | -10.33 | 27.95 | Yes | *** | -11.87 to -8.797 |
| Control vs PA  +  Lactobacillus | -3.833 | 10.37 | Yes | *** | -5.369 to -2.297 |
| Control vs PA  + Thymol | -3.333 | 9.017 | Yes | *** | -4.869 to -1.797 |
| Control vs PA  + COMB | -1.500 | 4.058 | No | ns | -3.036 to 0.03616 |
| PA vs PA +  Lactobacillus | 6.500 | 17.58 | Yes | *** | 4.964 to 8.036 |
| PA vs PA +  Thymol | 7.000 | 18.94 | Yes | *** | 5.464 to 8.536 |
| PA vs PA +  COMB | 8.833 | 23.89 | Yes | *** | 7.297 to 10.37 |
| PA +  Lactobacillus vs PA + | 0.5000 | 1.353 | No | ns | -1.036 to 2.036 |

| Thymol |  |  |  |  |  |
| --- | --- | --- | --- | --- | --- |
| PA +  Lactobacillus vs PA +  COMB | 2.333 | 6.312 | Yes | ** | 0.7972 to 3.869 |
| PA + Thymol vs PA +  COMB | 1.833 | 4.959 | Yes | * | 0.2972 to 3.369 |

Supplementary table 4 statistical values of 1^st^ day condition

| Tukey's Multiple Comparison Test | | | | | |
| --- | --- | --- | --- | --- | --- |
| 2^nd^ day  condition | Mean  Diff. | q | Significant?  P < 0.05? | Summary | 95% CI of diff |
| Control vs PA | -5.000 | 15.99 | Yes | *** | -6.299 to -3.701 |
| Control vs PA  +  Lactobacillus | -1.333 | 4.264 | Yes | * | -2.633 to -0.03398 |
| Control vs PA  + Thymol | -1.000 | 3.198 | No | ns | -2.299 to 0.2993 |
| Control vs PA  + COMB | -0.6667 | 2.132 | No | ns | -1.966 to 0.6327 |
| PA vs PA +  Lactobacillus | 3.667 | 11.73 | Yes | *** | 2.367 to 4.966 |
| PA vs PA +  Thymol | 4.000 | 12.79 | Yes | *** | 2.701 to 5.299 |
| PA vs PA +  COMB | 4.333 | 13.86 | Yes | *** | 3.034 to 5.633 |
| PA +  Lactobacillus | 0.3333 | 1.066 | No | ns | -0.9660 to 1.633 |

| vs PA +  Thymol |  |  |  |  |  |
| --- | --- | --- | --- | --- | --- |
| PA +  Lactobacillus  vs PA + COMB | 0.6667 | 2.132 | No | ns | -0.6327 to 1.966 |
| PA + Thymol  vs PA + COMB | 0.3333 | 1.066 | No | ns | -0.9660 to 1.633 |
